# Combined HAT/EZH2 modulation leads to cancer-selective cell death

**DOI:** 10.18632/oncotarget.25428

**Published:** 2018-05-22

**Authors:** Francesca Petraglia, Abhishek A. Singh, Vincenzo Carafa, Angela Nebbioso, Mariarosaria Conte, Lucia Scisciola, Sergio Valente, Alfonso Baldi, Amit Mandoli, Valeria Belsito Petrizzi, Concetta Ingenito, Sandro De Falco, Valeria Cicatiello, Ivana Apicella, Eva M. Janssen-Megens, Bowon Kim, Guoqiang Yi, Colin Logie, Simon Heath, Menotti Ruvo, Albertus T.J. Wierenga, Paul Flicek, Marie Laure Yaspo, Veronique Della Valle, Olivier Bernard, Stefano Tomassi, Ettore Novellino, Alessandra Feoli, Gianluca Sbardella, Ivo Gut, Edo Vellenga, Hendrik G. Stunnenberg, Antonello Mai, Joost H.A. Martens, Lucia Altucci

**Affiliations:** ^1^ Dipartimento di Medicina di Precisione, Università degli Studi della Campania Luigi Vanvitelli, Napoli 80138, Italy; ^2^ Department of Molecular Biology, Radboud University, HB Nijmegen 6500, The Netherlands; ^3^ IRCCS SDN, Napoli 80143, Italy; ^4^ Dipartimento di Chimica e Tecnologie del Farmaco ‘Sapienza’ Università, Roma 00185, Italy; ^5^ Dipartimento di Scienze e Tecnologie Ambientali, Biologiche e Farmaceutiche, Università della Campania ‘Luigi Vanvitelli’, Caserta 81100, Italy; ^6^ Ospedale Umberto I, Nocera Inferiore 84014, Italy; ^7^ Istituto di Genetica e Biofisica, Napoli 80131, Italy; ^8^ Centro Nacional de Análisis Genómico, Barcelona, Spain; ^9^ Istituto di Biostrutture e Bioimmagini, Napoli, Italy; ^10^ Department of Hematology, University of Groningen and University Medical Center Groningen, RB Groningen 9700, The Netherlands; ^11^ European Molecular Biology Laboratory, European Bioinformatics Institute, Wellcome Trust Genome Campus, Hinxton, United Kingdom; ^12^ Max Planck Institute for Molecular Genetics, Berlin, Germany; ^13^ Institute Gustave Roussy, Equipe labellisée Ligue Nationale contre le Cancer (LNCC), Universtité Paris-Saclay, INSERM U1170, Paris, France; ^14^ Dipartimento di Farmacia, Università di Napoli ‘Federico II’, Napoli 80131, Italy; ^15^ Dipartimento di Farmacia, Università degli Studi di Salerno, Fisciano I-84084, Italy; ^16^ Pasteur Institute, Cenci-Bolognetti Foundation, Sapienza University of Rome, Roma 00185, Italy

**Keywords:** cancer, epigenetics, apoptosis, acetylation, methylation

## Abstract

Epigenetic alterations have been associated with both pathogenesis and progression of cancer. By screening of library compounds, we identified a novel hybrid epi-drug MC2884, a HAT/EZH2 inhibitor, able to induce *bona fide* cancer-selective cell death in both solid and hematological cancers *in vitro*, *ex vivo* and *in vivo* xenograft models. Anticancer action was due to an epigenome modulation by H3K27me3, H3K27ac, H3K9/14ac decrease, and to caspase-dependent apoptosis induction. MC2884 triggered mitochondrial pathway apoptosis by up-regulation of cleaved-BID, and strong down-regulation of BCL2. Even aggressive models of cancer, such as p53^–/–^ or TET2^–/–^ cells, responded to MC2884, suggesting MC2884 therapeutic potential also for the therapy of TP53 or TET2-deficient human cancers. MC2884 induced massive apoptosis in *ex vivo* human primary leukemia blasts with poor prognosis *in vivo*, by targeting BCL2 expression. MC2884-treatment reduced acetylation of the BCL2 promoter at higher level than combined p300 and EZH2 inhibition. This suggests a key role for BCL-2 reduction in potentiating responsiveness, also in combination therapy with BCL2 inhibitors.

Finally, we identified both the mechanism of MC2884 action as well as a potential therapeutic scheme of its use. Altogether, this provides proof of concept for the use of epi-drugs coupled with epigenome analyses to ‘personalize’ precision medicine.

## INTRODUCTION

Massive parallel sequencing of cancer genomes has identified a myriad of mutant epigenetic enzymes responsible for histone acetylation and methylation, and DNA methylation [1]. Deregulation of the complex interplay between genome and epigenome thus provides a fertile ground for cancer development and progression [2], implicating epigenetic ‘writers’ [3–5], ‘erasers’ [5, 6], and ‘readers’ [7–12]. Since aberrant expression of epigenetic enzymes plays a causative role in tumorigenesis, the reversal of these modifications has emerged as a potential strategy for cancer treatment [13–15]. Genome sequencing of *de novo* AML revealed the presence of at least one non-synonymous mutation in 44% of DNA methylation-related genes and 30% of chromatin-modifying genes [1, 16]. This astonishing finding highlights the dual etiology - epigenetic and genetic - phenomena driving leukemogenesis [17, 18] and likely other cancers [19]. As a result, a number of therapeutic compounds targeting epigenetic enzymes have been developed [20–28]. However, because cancer relapse due to acquired resistance to treatments remains a major concern, novel classes of ‘epi-drugs’ are urgently needed. The evidence that histone methylation is deregulated in cancer, identified lysine methyltransferases and demethylases as potential targets for new anticancer drugs. Indeed, inhibitors targeting the methyltransferases DOT1L, EZH2 or the demethylase LSD1 are in clinical trials [29]. Whether more of these selective or more general chromatin regulators are needed is still under investigation [30]. In some cancers the presence of mutated chromatin enzymes has led to the development of epidrugs active preferentially on the mutant form. On the other hand, a broader acting drugs or hybrid molecule might prove more useful when concomitant alterations of different epi-targets are involved in the tumorigenic process. This might also apply to tumor heterogeneity.

Here, we identify and characterize a novel small molecule displaying inhibitory actions on both EZH2 and acetyltransferases. To the best of our knowledge this compound is the first displaying this hybrid activity in the low micromolar range. We characterize its anticancer action on a panel of cell lines, *in vivo* tumor models and *ex vivo* patient’s blasts. We identify the mechanism of anticancer action as causal activation of mitochondrial apoptotic players such as modulating the expression of BCL2 (and related homologues) at the chromatin level. We found that the new molecule is able to exert apoptosis in very aggressive models of cancer. We propose both diagnostic tools and potential therapeutic strategies to turn this knowledge into practice by using the new inhibitor together with available drugs.

## RESULTS

### MC2884 induces cancer-specific cell death

**Figure 1:**
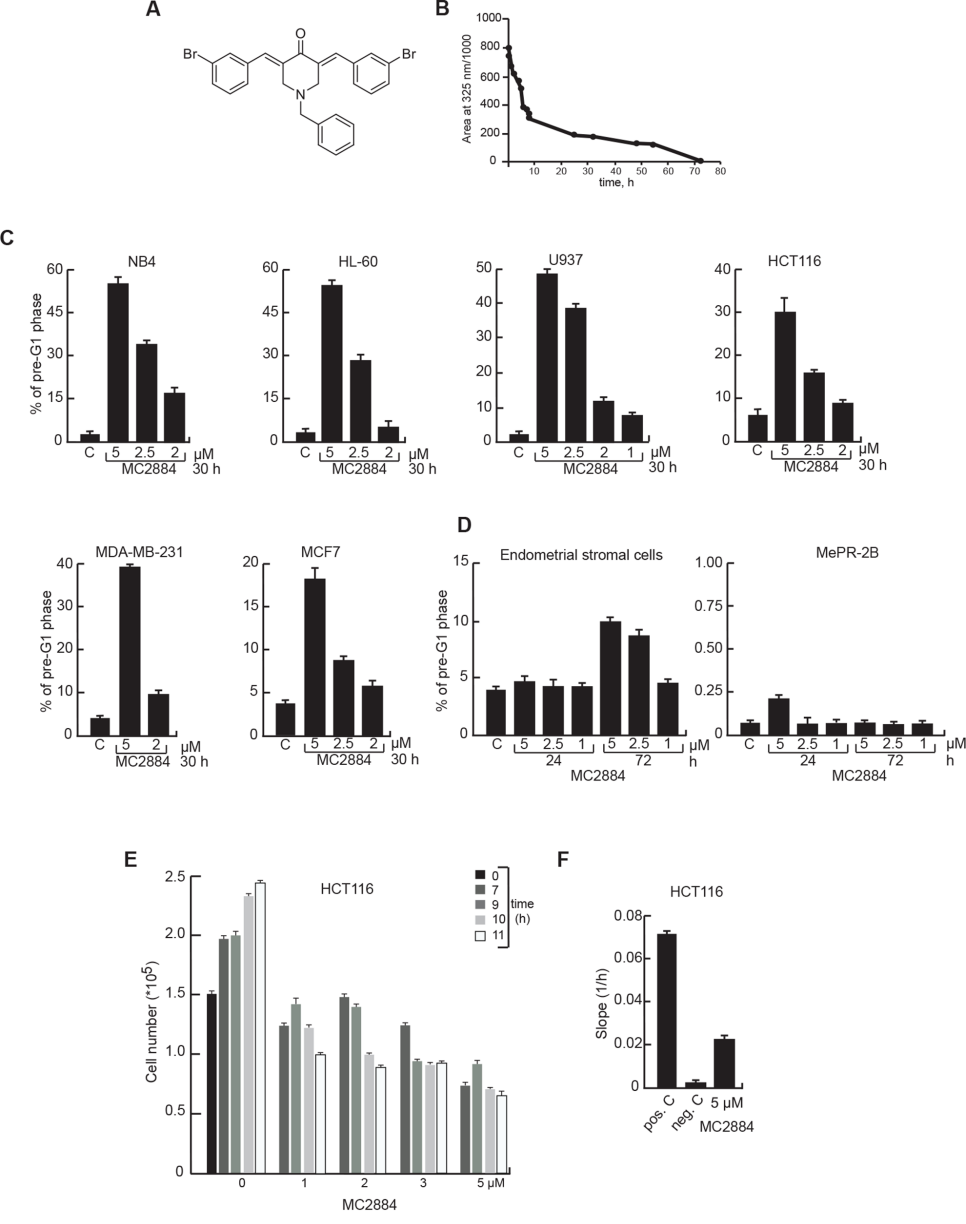
MC2884 induces time- and dose-dependent cancer cell apoptosis (**A**) Chemical structure and (**B)** half-life in culture medium of MC2884. (**C)** Cell death evaluation by FACS, induced by MC2884 in hematological and solid cancer cells, and (**D)** in normal cells at the indicated times and concentrations. (**E)** Anti-proliferative action of MC2884 in colon cancer cells measured in real time. (**F)** Migration inhibitory action of MC2884 measured in colon cancer cells as Slope (1/h). MC2884 was used at indicated time and concentrations. Curves and graph presented showed the mean of at least two different experiments with an error bars indicating standard deviation.

To identify novel epigenetic drugs with hybrid actions, we screened a panel of compounds of natural or synthetic origin harboring potential chromatin activity (see Supplementary Information, Chemistry and SAR studies) and identified MC2884 (Figure 1A) as a promising candidate. Its possible therapeutic use was supported by its high stability in cell culture media whereby it showed a half-life of 10 h and a time for total degradation of 72 h (Figure 1B). Based on structural modeling, MC2884 was suggested to act as a modulator of EZH2 activity [31]. MC2884 has profound anti-proliferative effects on a variety of cancers. As a first screen, we investigated the effects of MC2884 on cell death induction in several cancer cell lines derived from leukemias (NB4, HL-60, U937) or solid tumors, such as colon (HCT116) and breast (MDA-MB231, MCF7) (Figure 1C). Similar data were obtained in cervix (HeLa) and brain (Kelly, U87) cancer cell lines (Supplementary Figure 1A).

MC2884 induced dose-dependent cell death after a 30-hour treatment. While all cancer cell-lines respond to MC2884, leukemic cells, despite some differences depending on their origin, appear generally the most responsive. Next we evaluated the cytotoxic effect of MC2884 on two different immortalized non-cancer cell systems, endometrial stromal cells [32] and the mesenchymal progenitor model MePR2B [33, 34] (Figure 1D) revealing that both ‘normal cell’ models were insensitive to the treatment. These data indicate that inhibitory action of MC2884 on the cell growth is potentially tumor selective.

To assess whether and when MC2884 would affect both proliferation and invasiveness of cancer, we performed proliferation and migration analyses using HCT116 cells in real-time mode treated with different concentrations (1 to 5 μM) (Figure 1E–1F). An inhibitory effect of MC2884 on proliferation was already evident at early time points (7–9 h), suggesting that the anti-proliferative action of MC2884 is early occurring. Similar data were obtained in NB4 cells (Supplementary Figure 1B–1C). The migration inhibitory effect of MC2884 evaluated by slope analysis [35] showed a strong decrease of migration rate after 24 h of treatment (Figure 1F), suggesting a strong anti-invasion effect of the drug. These data strongly imply wide-ranging anti-proliferative, anti-invasiveness and apoptotic effects of MC2884 in low micromolar ranges.

### MC2884 PRC2 and HAT inhibitory activities contribute to its anticancer effects

**Figure 2:**
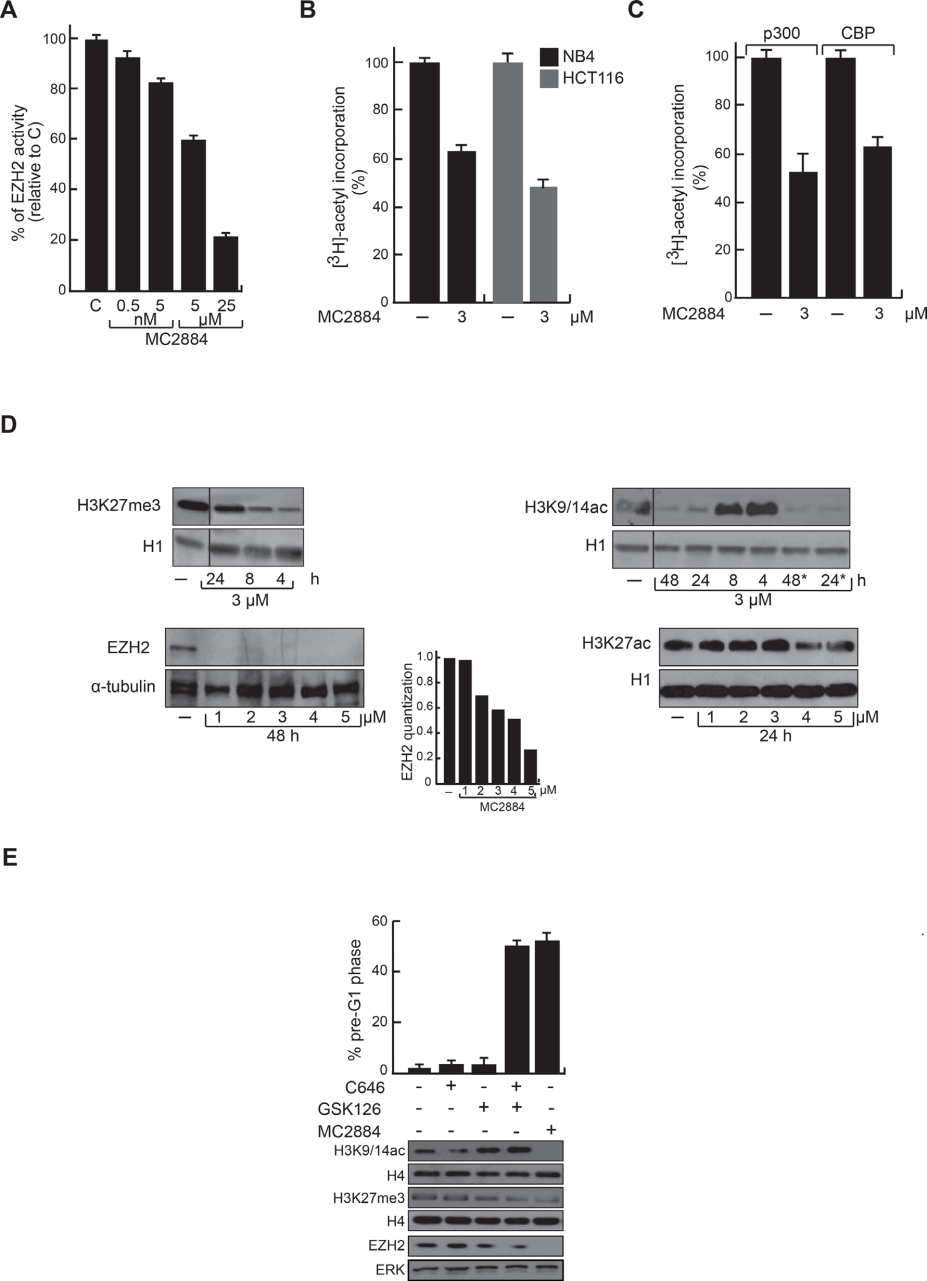
Chromatin modulating effects of MC2884 *in vitro* and in cancer cells (**A)** MC2884 inhibits EZH2 activity *in vitro*. (**B)** MC2884 inhibits HAT activity in total cell extracts. (**C)** MC2884 inhibits the indicated HATs *in vitro*. (**D)** Western blot analyses for H3K27me3, H3K27ac, EZH2 and H3K9/14ac indicate that MC2884 is able to modulate their expression in NB4 APL cells. ^*^indicates that the MC2884 has been added a second time after 10 h. Histone H1 and tubulin represent loading controls. (**E)** Simultaneous but not single inhibition of EZH2 and p300 displays anticancer effect in NB4 APL cells, as shown by FACS analysis (top). Western blot analyses for H3K9/14ac, H3K27me3 and EZH2 showing the efficacy of the indicated treatments (bottom). MC2884 was used at indicated time and concentrations. Graph showed the mean of three independent experiments with error bars indicating standard deviation.

To decode the basis of MC2884 anticancer effects, the methyltransferase activity of EZH2 was evaluated in the presence of MC2884. *In vitro* assays showed that MC2884 is a genuine inhibitor of the EZH2 enzyme in a dose-dependent manner, reaching values of inhibition of about 40% and 80% at 5–25 μM, respectively (Figure 2A). To address the MC2884 inhibitory action on histone acetylation, HAT assays were performed on total cell extracts in both NB4 and HCT116 (Figure 2B), showing that MC2884 decreased histone acetyltransferase activity in living cells. In addition, MC2884 was able to decrease the acetyltransferase activity of immunoprecipitated p300 and CBP (Figure 2C), suggesting a HAT inhibition at low micromolar range. Modulation of the methyltransferase and HAT inhibitory action was time and dose dependent as corroborated by H3K27me3, H3K27ac and H3K9/14ac decrease (Figure 2D and Supplementary Figure 2A) as well as by the reduction of EZH2 at RNA and protein levels (Figure 2D and Supplementary Figure 2B). Note that when tested for HDAC or SirT modulating action, MC2884 showed no activity (Supplementary Figure 2C), strengthening its specificity towards EZH2 and HATs. To examine whether both epigenome targeting actions contribute to MC2884 anticancer effects, we used the p300 inhibitor C646 and the EZH2 antagonist GSK126, either separately or in combination. Our analysis revealed the compounds had a synergic effect on cell death, while no effects were seen when using the single inhibitors (Figure 2E). Taken together these results suggest that MC2884 modulates the epigenome through inhibition of EZH2 and HAT actions in the low μM range giving rise to decreased histone acetylation and H3K27me3 in different cancer cell lines.

### MC2884 induces caspase- and mitochondrial-dependent cancer-selective apoptosis

**Figure 3:**
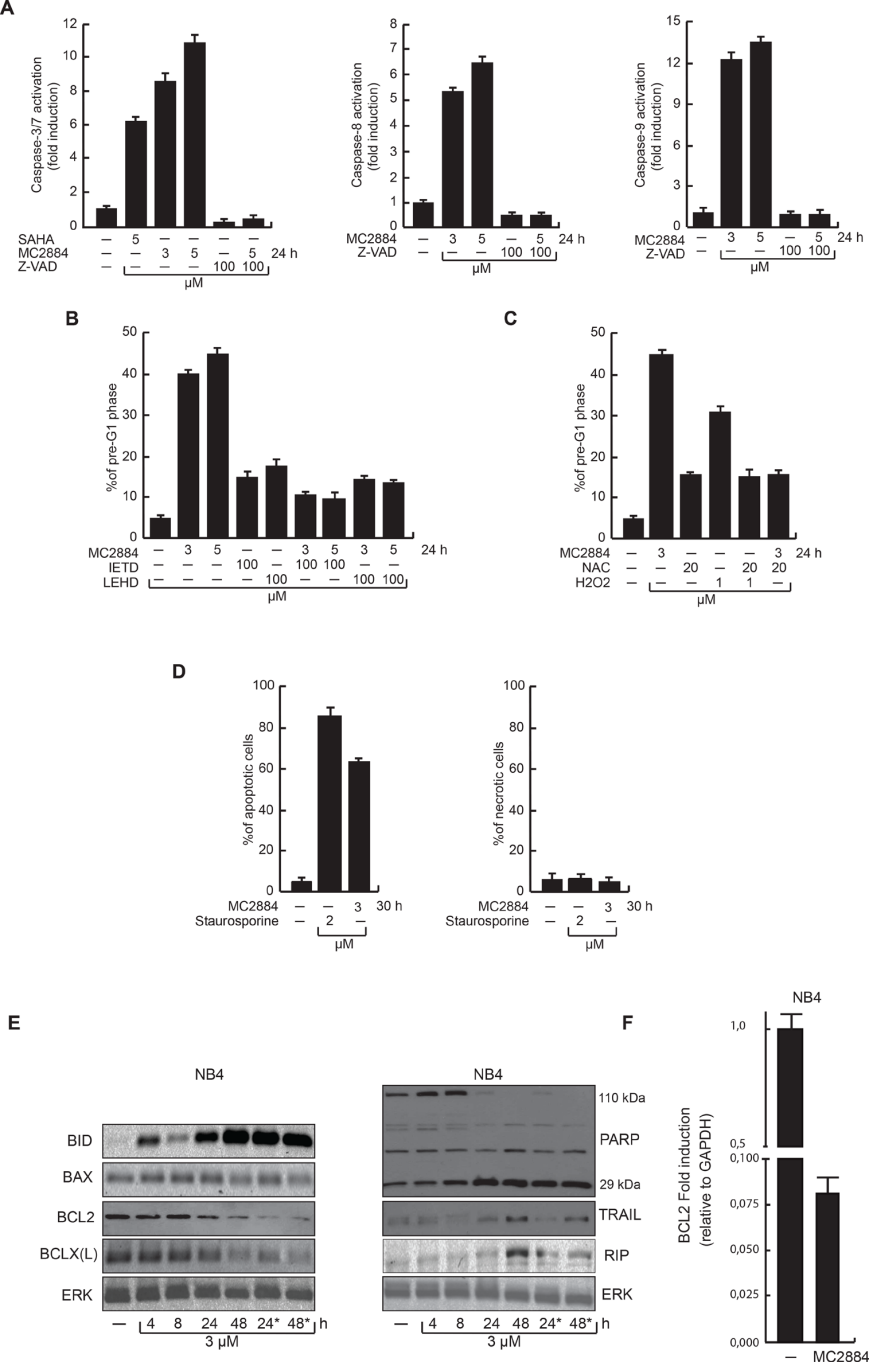
MC2884 induces caspase-dependent apoptosis accompanied by ROS production (**A)** FACS analyses of caspase 3/7, 8 and 9 activation upon MC2884 and caspase inhibitors as indicated. SAHA has been used as positive control. (**B)** FACS analysis of pre-G1 phase in NB4 cells upon treatment with MC2884 in presence of the Caspase 8 and 9 inhibitors, as indicated. (**C)** FACS analysis of pre-G1 phase in NB4 cells in presence of NAC at the indicated concentration. H_2_O_2_ has been used as positive control. (**D)** FACS analysis of apoptosis (left) and necrosis (Annexin/PI) (right) assays following treatment with MC2884, staurosporine or vehicle for 30 h in NB4 cells. (**E)** Western blot analyses for cleaved BID, BAX, BCL2, BCLX(L), PARP, TRAIL and RIP expression levels in NB4 treated with MC2884 at the indicated time. ^*^indicates that the MC2884 has been added a second time after 10 h. ERK has been used as reference for loading; (**F)** RT-PCR analysis of BCL2 RNA expression levels in NB4 treated with MC2884 for 48 hours. MC2884 was used at indicated time and concentrations. Graph showed the mean of three independent experiments with error bars indicating standard deviation.

To gain mechanistic insight into the cell death induced by MC2884, caspase-3/7, -8 and -9 activities were analyzed in leukemic NB4 cells. MC2884 treatment induced caspase-3/7 activity in a dose-dependent manner as well as the activation of both caspase-8 and -9 (Figure 3A). This activation was blocked by the pan-caspase inhibitor Z-VAD, showing the key role of caspases in MC2884-mediated apoptosis (Figure 3A). To support these findings, we examined cell death in NB4 upon the co-treatment of MC2884 with IETD and LEHD that specifically inhibit caspase-8 and 9, respectively. This analysis corroborated and extended our finding that MC2884-mediated apoptosis is fully caspase-dependent (Figure 3B). Preliminary evidence of mitochondrial damage by caspase-9 activation suggested the induction of oxidative stress in cells treated with MC2884. To investigate this further, we treated NB4 cells with MC2884 and the antioxidant N-acetylcysteine (NAC). NAC was able to block apoptosis induced by MC2884, indicating that MC2884-induced apoptosis is associated with ROS production, which is causally related to the mechanism of cell death (Figure 3C). Data with double Annexin V-propidium iodide (PI) staining strongly confirmed that MC2884, like staurosporine, only induced apoptosis cell death (Figure 3D).

To further identify and characterize the molecular pathways affected by MC2884, protein levels of crucial players of the apoptosis pathway were investigated in NB4 cells (Figure 3E). MC2884 induced activation of the mitochondrial pathway by up-regulation of cleaved-BID, and strong down-regulation of BCL2 (Figure 3E–3F) and BCL2-XL. In accordance with caspase-3/7 activation, MC2884 was able to induce activation of PARP, in particular after 24 h. The fact that TRAIL regulation, if at all, was weak, whereas RIP up-regulation was relatively late occurring, suggests that especially the intrinsic pathways associated with caspase-9 and apoptosome activation may play an essential role in cancer cell death induction. Taken together, this data indicates that MC2884 is able to induce strong apoptosis by activating caspase-dependent pathways.

### MC2884 displays anticancer action in both hematological and solid cancer *in vivo* models

**Figure 4:**
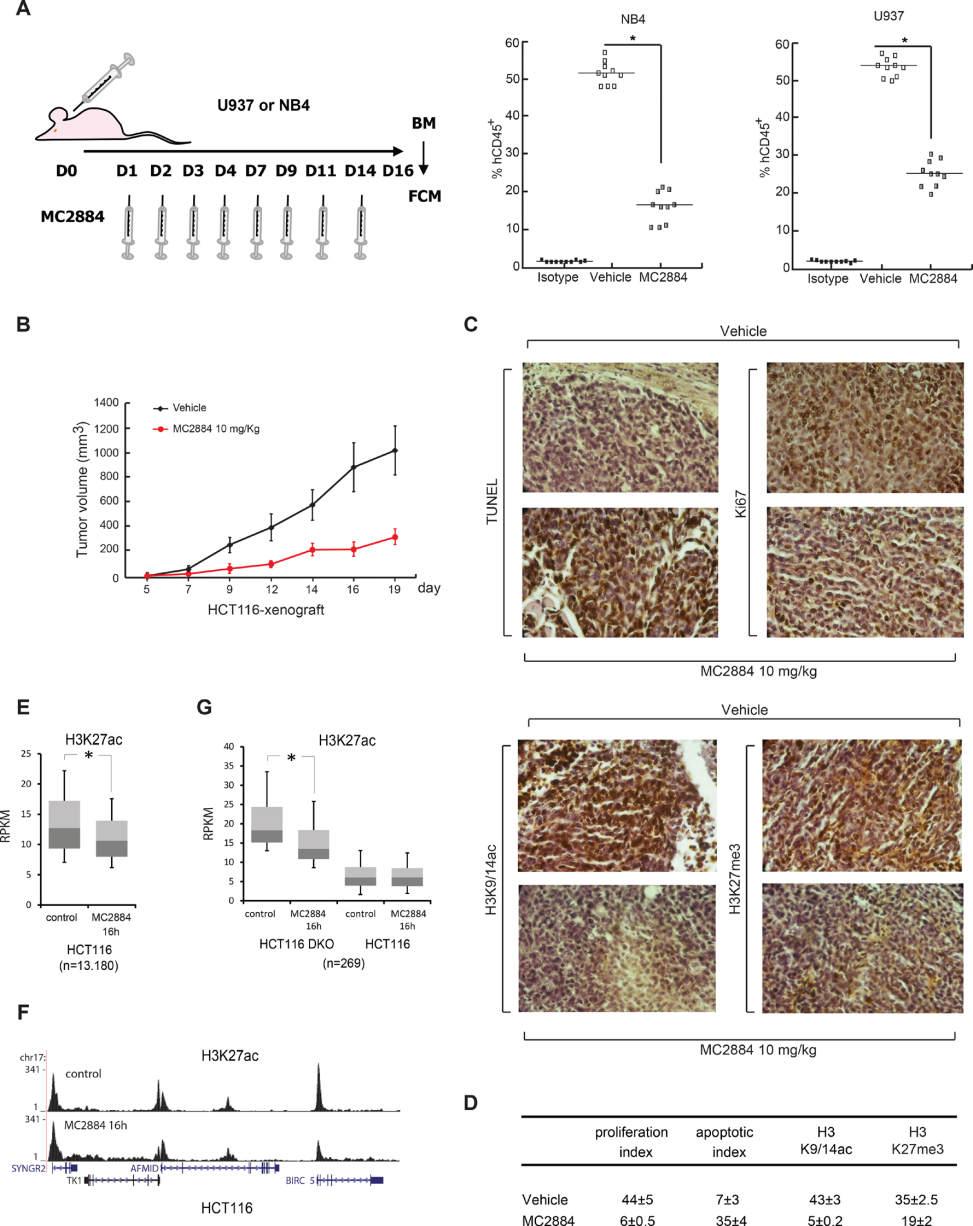
MC2884 displays anticancer action in both hematological and solid cancer *in vivo* (**A)** *In vivo* experimental design for leukemia xenografts treated with MC2884 (1 mg/Kg). At day 16, bone marrow (BM) cells were isolated. MC2884 antiproliferative action was evaluated by FACS analysis of the % of human CD45+ cells in the isolated population. (**B)** Colon cancer (HCT116) xenograft. *In vivo* growth expressed in volume of tumors induced. (**C)** Immunohistochemical analyses and statistical evaluation for the apo-index, Ki67, H3K9/14ac, H3K27me3 showed significant alterations (increased apo-index and decreased Ki67, H3K9/14ac, H3K27me3) in MC2884-treated versus control (vehicle) cells (*p* = 0.002). (**D)** Ki67 (proliferation index) and TUNEL (apoptotic index) scores were analyzed at the end of treatment. The proliferation index was significantly lower in tumors of treated mice compared to controls (*p* = 0.002). Apoptotic index was significantly higher in tumors of MC2884-treated mice (*p* = 0.002). (**E)** Boxplot of H3K27ac levels at hyperacetylated regions in HCT116 cells before and after treatment for 16 h with MC2884; (**F)** H3K27ac levels at the BIRC5 locus before and after treatment with MC2884; (**G)** Boxplot of H3K27ac levels at hyperacetylated regions in HCT116-DKO cells before and after treatment for 16 h with MC2884. Curves and graph presented showed the mean of at least two different experiments with an error bars indicating standard deviation.

To assess the anti-leukemic activity of MC2884 *in vivo*, immune-compromised mice were retro-orbitally injected with U937 or NB4 cells, and were treated three times weekly with intra-peritoneal administration of MC2884 1 mg/kg (Figure 4A, left). The mice were sacrificed 16 days post-xenograft and displayed a normalized weight of 94%, and 90% of their initial weight, for control mice and MC2884-treated mice, respectively, suggesting that the MC2884 was well tolerated in these settings. The bone marrow of each mouse was examined by flow cytometry and human NB4 and U937 xenografted cells were discriminated by human CD45^+^ versus mouse CD45^+^ staining. This *in vivo* experiment demonstrated the high anti-proliferative activity of MC2884 in both leukemic models: in NB4 cells human CD45^+^ cells reduced to 17%, while in U937 to 24% in the bone marrow (Figure 4A, right), suggesting a strong *in vivo* anti-leukemic action. These results could be further strengthened using NB4 cells expressing luciferase (Supplementary Figure 3A). The fact that NB4 cells allowed to grow for 5 days prior to the start of drug treatment still responded similarly to MC2884 suggested that there was no major impact on homing and engraftment due to time of cell and drug injection (Figure 4A, right).

MC2884 also displayed a strong anticancer effect in a xenograft model of HCT116 colon carcinoma. Mice treated with 10 mg/Kg MC2884 showed a markedly reduced cancer growth as highlighted by a decrease in tumor mass (Figure 4B). Notably, this MC2884-mediated tumor volume reduction was 90% of that of untreated mice on day 19. In line with this effect, immunohistochemical analyses showed that MC2884 was able to induce strong apoptosis as revealed by TUNEL assays, and a block of cell proliferation as revealed by Ki67 staining (Figure 4C, top). In addition, and corroborating the effect on the epigenome of MC2884 obtained in cancer cell lines, MC2884 displayed a strong down-regulation of both H3K9/14ac and H3K27me3 as shown (Figure 4C, bottom) and statistically quantified (Figure 4D). Similar effects were observed in a HCT116^p53−/−^ xenograft model (Supplementary Figure 3E).

In a separate analysis, we examined genome-wide H3K27ac in HCT116 cells and observed a decrease after treatment with MC2884 (Figure 4E), for example at the anti-apoptotic gene BIRC5 (Figure 4F). In addition, when using DNMT1/DNMT3B double knock out HCT116 cells (HCT116 DKO), which are characterized by increased acetylation [36], we again observed decreases in H3K27ac after MC2884 treatment specifically at those regions hyperacetylated in HCT116 DKO as compared to normal HCT116 (Figure 4G; see Supplementary items for GO analysis). These results strengthen MC2884 anticancer action in both hematological and solid cancer *in vivo* models and its connection to epigenomic modulation.

### MC2884 induces apoptosis in aggressive cancers and in primary leukemic blasts

**Figure 5:**
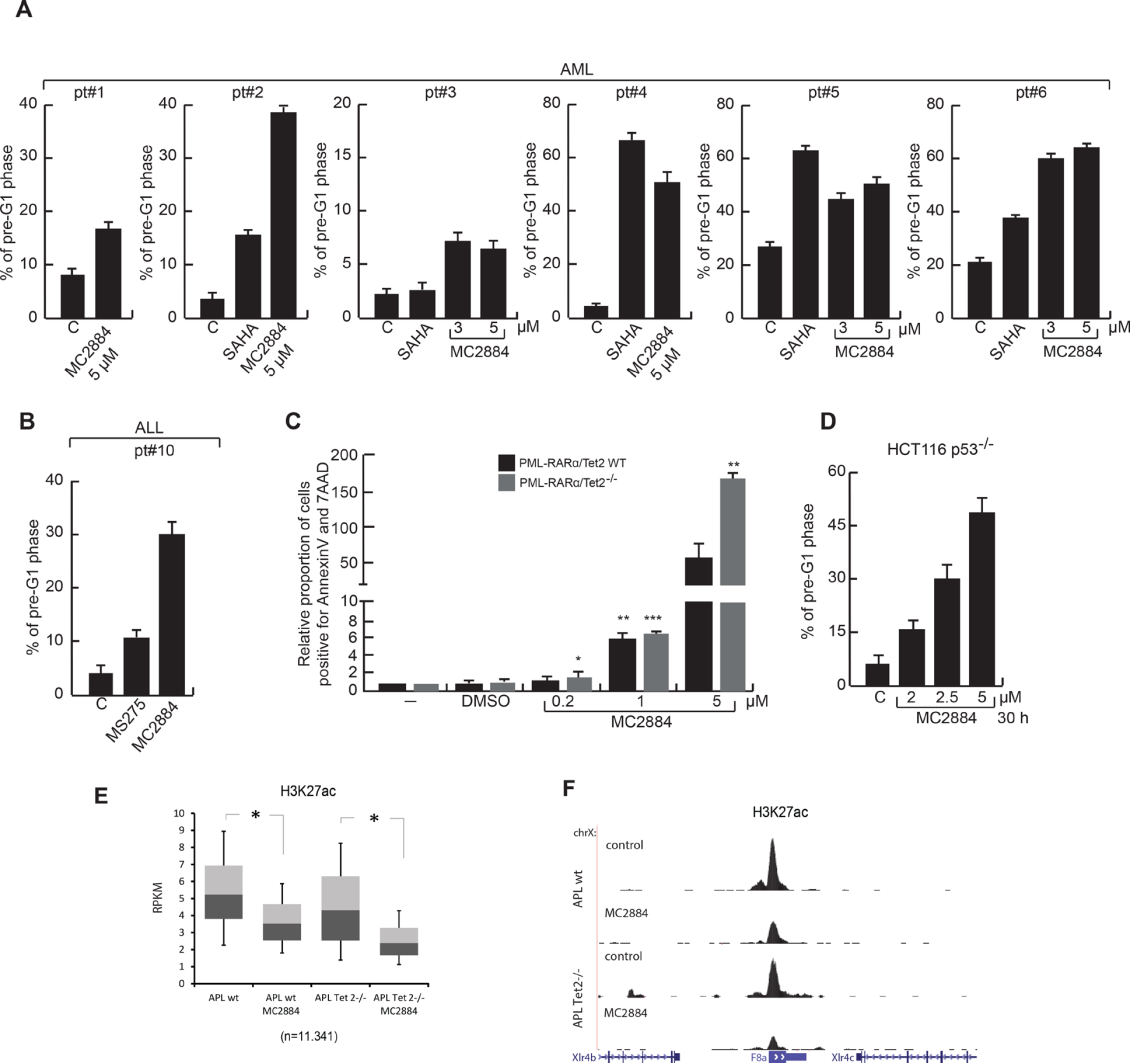
MC2884 induces anticancer action in *ex vivo* AMLs, ALL and aggressive models of cancer (**A**–**B)** FACS analysis of cell death induced by MC2884, HDACi (MS275 and SAHA) and ATRA in *ex vivo* AML and ALL (24 h). (**C)** Analysis of apoptosis in APL-Tet2WT and APL-Tet2^−/−^ cells upon treatment with various concentration of MC2884. Shown is the proportion of dying cells, normalized to non-treated conditions. Statistical significance is indicated by stars; (**D)** FACS analysis of cell death induced by MC2884 in HCT116 p53–/– colon cancer cells. (**E)** Boxplot of H3K27ac levels at hyperacetylated regions in APL-wt (left) and APL-Tet2–/– (right) cells either not (control) or MC2884 treated. (**F)** ChIP-seq analysis of H3K27ac levels at two genomic loci in APL-wt (top) and APL-Tet2−/− (bottom) cells either not (control) or MC2884 treated. MC2884 was used at indicated time and concentrations. Graph showed the mean of three independent experiments with error bars indicating standard deviation

To further strengthen our findings in primary human cells, cell death was evaluated in 6 AML patient blasts (Figure 5A). In all cases, 24-hour treatment with MC2884 correlated with induction of cell death, revealed as percentage of cells in pre-G1 phase, to similar or even higher levels as with SAHA and MS275, used as cell death positive controls. In addition, MC2884 treatment led to death in *ex vivo* primary blasts derived from one acute lymphoblastic leukemia (ALL) (Figure 5B). Accordingly, MC2884 was able to induce an apoptotic response in Tet2^−/−^ APL murine cells, a mouse model for aggressive human APL (Figure 5C), and in HCT116-^p53−/−^ colon cancer cells (Figure 5D), which also displays a very aggressive behavior, suggesting MC2884 therapeutic potential also for TP53 or TET2-deficient human cancers.

In full agreement with our findings, H3K27ac ChIP-seq analysis of MC2884 treated APL-Tet2^−/−^ and APL-wt murine cells showed a strong H3K27ac reduction in both (Figure 5E–5F; see Supplementary items for GO analysis).

### MC2884-treatment targets BCL2 and synergizes with BCL2 inhibitors

**Figure 6:**
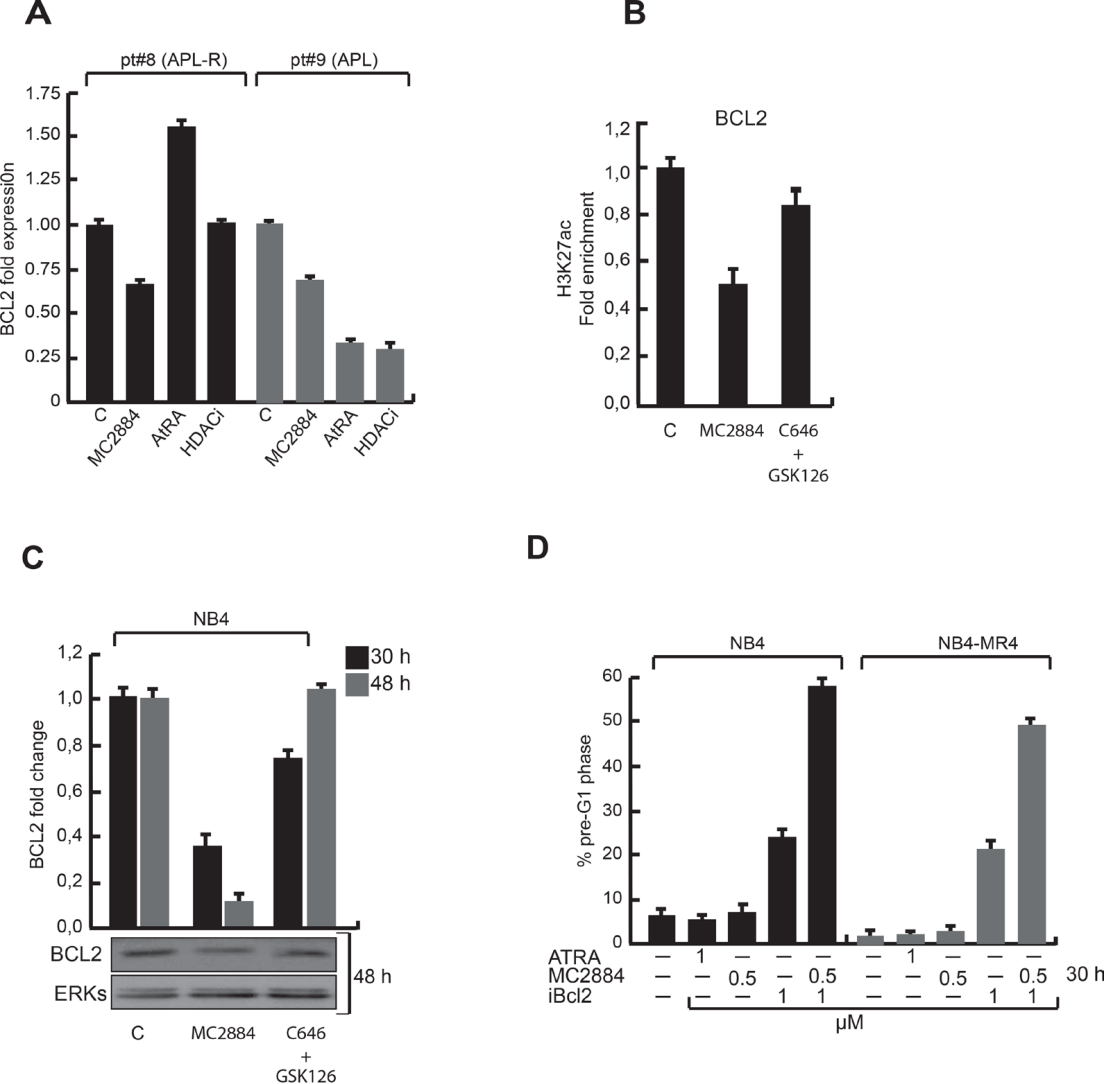
Epigenetic changes upon MC2884 treatment result in reduced BCL2 expression **(A)** BCL2 expression by RT-qPCR in acute promyelocityc leukemia sensitive and resistant to standard treatments. (**B)** ChIP in NB4 cells using H3K27ac antibodies and primers for the BCL2 promoter with the indicated treatments. (**C)** BCL2 expression by RT-qPCR (top) and western (bottom) after the indicated treatments. (**D)** FACS analysis of % of AtRA responsive (NB4) and AtRA resistant (NB4-MR4) cells in pre-G1 upon the indicated treatments. MC2884 was used at indicated time and concentrations. Graph showed the mean of three independent experiments with error bars indicating standard deviation.

In agreement with our earlier results (Figure 3D–3E), decreased BCL2 RNA expression was observed upon MC2884 treatment in primary blasts derived from an APL sample derived from a high risk APL (pt#8) (Figure 6A) described in the accompanying manuscript. Inversely, ATRA induced an up-regulation of BCL2. Notably, in an ATRA sensitive APL sample (pt#9), ATRA induced a down-regulation of BCL2, suggesting a key role for BCL-2 reduction in allowing good responsiveness.

Importantly, MC2884-structurally related compounds inactive in inducing both anticancer effects and epigenome modulation were not able to down-regulate BCL2 (see Supplementary SAR studies). In line with reduced BCL2 expression, MC2884 reduced BCL2 promoter acetylation (Figure 6B). Moreover it decreased BCL2 expression more than a p300 and EZH2 inhibitor combination (Figure 6C), suggesting the combination of the epigenetic inhibitory activities in one molecule makes it more potent.

To assess the contribution of BCL2 to the antitumor action of MC2884, the effects of an inhibitor of BCL2 (ABT-737) were analyzed (Figure 6D). The anti-leukemic action of ABT-737 was strongly potentiated, reaching up to 60% of cell death, by addition of MC2884 at concentrations that alone induced little apoptosis. This synergistic antitumor effect was also reproduced in the resistant NB4-MR4 [37], suggesting that MC2884 has potential for combination therapy in a wide range of leukemias.

## DISCUSSION

Currently approved treatments focusing on deregulation of epigenetic components are often based on the use of HDACi [38, 39] or DNA demethylating agents, aiming at chromatin opening by increasing acetylation levels or lowering DNA methylation, respectively. However, conceptually many pathways that increase stemness and cancer cell survival should rather be switched off. We here describe such an opposite approach and as such a potential ‘paradigm shift’, where hyperacetylation of chromatin areas together with selective H3K27 methylation (and consequently transcriptome and methylome remodeling) are targets for cancer therapy.

MC2884, selected from a collection of newly synthesized chromatin-modulating drugs for its strong anticancer potential, seems highly effective in the treatment of solid and hematopoietic cancers, including relapsed cancers and those that are resistant to standard treatments. It unbalances histone acetylation acting as a *bona fide* HAT inhibitor in the low micromolar range. Displaying also an inhibitory action towards EZH2, it potentially targets and counteracts both pathologically opened and activated loci by inhibiting the acetyltransferases, and regions closed by increased H3K27me3 by the histone methyltransferase EZH2. MC2884 results in down-regulation of anti-apoptotic drivers such as BCL2 and BCLX(L), mitochondrial modulation and in activation of related death pathways that kill cancer cells in a selective manner. *In vivo*, AML, APL and colon cancer xenograft models suggest a very robust anticancer potential, which is most likely related to chromatin modulation. Excitingly, both TP53^−/−^ colon cancer and Tet2^−/−^ APL cells, which show a highly aggressive behavior [40–42], displayed high sensitivity to MC2884, suggesting that as a consequence of the MC2884-mediated induction of hypoacetylation, patients with aggressive cancer phenotypes (that may be in an overall hyperacetylated state) might benefit from this type of approach.

The mechanism of apoptosis induction by MC2884 treatment pointed to BCL2 mediated regulation; acetylation at BCL2 chromatin regions is reduced in MC2884-treated hrAPLs and in accordance BCL2 expression is decreased. Furthermore, BCL2 is oppositely regulated by ATRA in ‘normal’ APLs and hrAPLs being down- and up-regulated, respectively, suggesting a link to ATRA (and HDACi) resistance. Strikingly, the use of the BCL2 protein inhibitor (ABT-737) together with the chromatin modulation exerted by MC2884 on BCL2 is a successful combination that synergistically kills both ATRA-sensitive and -resistant APL cells. This suggests that hrAPL and other patients might benefit from a personalized, therapeutic strategy. Since the BCL2i ABT-737 is in clinical trial for different types of cancers [43], this approach should be feasible in not too long a time.

## MATERIALS AND METHODS

### Chemicals

SAHA (Merck), MS-275 (Alexis) and MC2884 were dissolved in dimethyl sulfoxide (DMSO) (Sigma); ATRA was obtained from Sigma.

### Chemistry

For general procedures see Supplementary Information.

### Stability of MC2884 in cell culture

The compound was dissolved in DMEM at 50 µM and its concentration was measured by RP-HPLC using a ONYX 50 × 2 mm ID C18 column operating at 600 µL/min. The gradient applied was from 1% solvent B to 70% solvent B in 10 minutes. Solvent A was water with added 0.1% trifluoroacetic acid (TFA) and solvent B was acetonitrile with added 0.1% TFA. The eluate was monitored using a diode array detector with wavelengths set between 200–320 nm. The compound was eluted at 11.1 min. Peak integration was carried out on the chromatogram extracted at 326 nm, which is one of the compound absorbance maxima. A calibration curve was built by injecting solutions of the compound in DMSO at concentrations ranging between 1.5 µM and 200 µM. After incubation in DMEM compound concentration was determined at time points ranging between 0 and 72 hrs. As shown, half of compound was degraded after 10 h. At 72 hrs it was fully degraded under these conditions.

### Cell culture

U937, NB4, HL-60, MCF7, MDA MB-231, HCT116, HeLa, Kelly and U87 tumor cell lines were purchased by DSMZ (NB4) and American Type Culture Collection (ATCC). Cell lines have been tested and authenticated following manufacturer’s instruction. All cell lines were maintained in an incubator at 37° C and 5% CO2. The human leukemia cells were grown in RPMI-1640 (Sigma-Aldrich) while human breast cancer cells in Dulbecco’s Modified Eagle Medium (DMEM) (Sigma) culture media, in presence of phenol red (GIBCO), 1% L-glutamine (EuroClone), 10% heat-inactivated Fetal Bovine Serum (FBS) (Sigma) and antibiotics. Endometrial Stromal Cells (ESC) were grown in DMEM-F12 culture medium with 10% FBS, 2mM L-glutamine and antibiotics. MePR2B were grown as previously reported [33].

### Cell proliferation, cell cycle and cell death analyses

For colorimetric exclusion the cells (2 × 10^5^ cells/mL) were plated in multiwells in triplicate. After stimulations at different times and concentrations, cells were diluted in the ratio 1:1 in Trypan Blue (Sigma) and counted with an optical.

For cell cycle analyses, cells were plated (2 × 10^5^ cells/mL) and after stimulation (performed as indicated in the text) were harvested, centrifuged at 1200 rpm for 5′ and resuspended in 500 μL of a hypotonic solution containing 1X PBS, Sodium Citrate 0.1%, 0.1% NP-40, RNAase A and 50 mg/mL Propidium Iodide (PI). After 30’ at room temperature (RT) in the dark, samples were acquired by FACS-Calibur (BD Bioscences, San Jose, CA, USA) using CellQuest software (BD Biosciences). The percentage in different phases of the cell cycle was determined by ModFit LT V3 software (Verity). All experiments were performed in triplicate.

Cell death was measured as percentage of cells in pre-G1 phase as in [44] Apoptosis vs necrosis was measured using apoptosis/necrosis kit as suggested by the supplier (Enzo life sciences).

### Proliferation and migration analysis in real time

Proliferation and migration analysis were performed by xCELLigence technology (Roche) following standard procedures and as reported in [35].

### Enzymatic assays

EZH2 assays were performed following BPS Biosciences instructions and as previously described [31].

Cell-based HAT assay. Protein extracts from the indicated cells were obtained in TAP buffer (Tris HCl pH 7.0 50 mM, NaCl 180 mM, NP-40 0.15%, glycerol 10%, MgCl_2_ 1.5 mM, NaMO_4_ 1 mM, NaF 0.5 mM) with protease inhibitor cocktail (Sigma), DTT 1 mM and PMSF 0.2 mM). Transfection with pCMX-Flag CBP and pP300 were performed in HEK293-FT cells and then proteins extracted. 1000 µg were diluted in TAP buffer and IPed with anti-p300 (Millipore, 2 µg) and anti-CBP (Santa Cruz, 2 µg) following standard procedures. As a negative control, purified IgG rabbit (Santa Cruz, 2 µg) and IgG mouse (Santa Cruz, 2 µg) were also used. All samples were washed in HAT Assay Buffer 1X (DTT 1 mM in PBS 1X) and the HAT radioactive assay was carried out. According to supplier’s instructions (Millipore), 10 µL of HAT Assay Buffer, 3 µL (2 µg) of Core Histones and 5 µL of the diluted [^3^H]-Acetyl-CoA were added to the beads. MC2884 was tested at the final concentration of 3 µM. All the components were incubated for 60 minutes at 37° C in gently rock/shake. Samples were spotted on P81 paper, washed three times with 10% trichloroacetic acid and once with acetone, transferred to a scintillation vial containing 5 mL scintillation cocktail and read in a scintillation counter. Anacardic Acid (AA, ENZO LIFE) was used directly on incubation mix, as positive control.

For HAT *in vitro* assay, MC2884 activity was also tested *in vitro* and IC50 was calculated as indicated in the scheme. MC2884 was tested in a 10-dose IC50 mode with 2-fold serial dilution, in singlet, starting at 200 μM. Anacardic Acid or C646, were tested in 10-dose IC50 mode with 3-fold serial dilution starting at 100 μM. Reactions were carried out at 3 μM Acetyl-CoA. Empty cells indicate no inhibition or compound activity that could not be fit to an IC50 curve.

**Table:** 

Acetyltransferase: Substrate:	Compound IC50(M)				
	CBP	GCN5	KAT5	p300	pCAF
	Histone H3	Histone H3	Histone H2A	Histone H3	Histone H3
**MC2884**	**3,27E-06**		**8,35E-07**	**4,56E-07**	
**C646**	**7,87E-08**		**2,04E-05**	**1,54E-07**	
**Anacardic Acid**		**3,86E-05**			**3,88E-05**

The HDAC1 and SIRT1 assays were performed as described previously [45, 46].

### Transfections

MCF7 cells were transfected with lipofectamine (Invitrogen) as previously described [47]. The following plasmids were used: 1245 pCMVb p300 was a gift from William Sellers (Addgene plasmid # 10717), MSCV-FlagmuEzh2deltaset-Hygro was a gift from Tobias Neff (Addgene plasmid # 49403), pCMVβ-p300. DY-myc was a gift from Tso-Pang Yao (Addgene plasmid # 30490) and pCMV-VSV-G-Ezh2 wt was a gift from Kristian Helin (University of Copenhagen). After induction with MC2884, cells were diluted 1:1 in Trypan blue (Sigma) and counted.

### Total protein, histone extraction and western blot analyses

The procedures were performed as described in [44, 48]. Primary antibodies used were: H3K27me3 (Abcam); H3K9-14ac (Millipore); H3K27ac, EZH2, PARP, TRAIL, RIP (Abcam); BID, BAX, BCL2 (Cell Signaling); BCL-XL (BD Biosciences); ERKs (Santa Cruz) and α-tubulin (Sigma) were used to normalize the total protein extracts, while H1 (Abcam) to normalize histone extracts.

### Caspase assays

Caspase activity was detected within living cells using B-BRIDGE Kits with cell-permeable fluorescent substrates following manufacture’s instructions. The fluorescent substrates for caspase-3, 7, -8, and -9 were FAM-DEVD-FMK, FAM-LETD-FMK, and FAM-LEHDFMK, respectively. Cells were washed twice in cold PBS and incubated for 1 h in ice with the corresponding substrates, as recommended by suppliers. Cells were analysed using Cell Quest software applied to a FACScalibur (BD Biosciences). Experiments were performed in triplicate and values expressed in mean ± SD.

### Reagents

Z-VAD (R&D), Caspase-8 Inhibitor Z-IETD-FMK (R&D) and Caspase-9 Inhibitor Z-LEHD-FMK (R&D) were used at 100 µM respectively. N-Acetyl cysteine (NAC) (Sigma) was used at 20 µM. ABT-737 (BCL2i) (Selleckchem) was used at 1 µM; GSK126 (EZH2i) (Selleckchem) was used at 10 µM; C646 (HATi) (Sigma) was used at 5 µM.

### RNA extraction and RT-PCR

Total RNA was extracted with Trizol (Invitrogen) and converted into cDNA using SuperScript^®^ VILO™ cDNA Synthesis Kit (Invitrogen) as described by supplier. RT-PCR was performed with 10–100 ng of cDNA template in a 25 μl total reaction volume (12.5 μl Bio-Rad iTaq Universal SYBR Green supermix (2×), 0.5 mM of each gene-specific primer, H_2_O up to volume). Reactions were carried out on a Bio-Rad CFX-96 real-time PCR system. Each reaction was run in triplicate.

For amplification the following primers were used: EZH2, forward (5′-CATCATAGCTCCAGCTCCCG-3′) and reverse (5′-CATCCCGGAAAGCGGTTTTG-3′); EED, forward (5′-CGATTTGCGACAGTGGG-3′) and reverse (5′-CAGGTGCATTTGGCGTG-3′); SUZ12, forward (5′-GTCCTGCTTGTGAAAGTTTGC-3′) and reverse (5′-CAAATGTCTTTTCCCCATCCT-3′); BCL2, forward (5′-GAACTGGGGGAGGATTGTGG-3′) and reverse (5′-CAGCCTCCGTTATCCTGGAT-3′); GAPDH, forward (5′-TCAACGGGAAGCCCATCACCA-3′) and reverse (5′-ACGGAAGGCCATGCCAGTGA-3′).

### Xenograft *in vivo* experiments

#### AML xenografts

Xenograft studies using NB4 and U937-AML cells were performed in NOG SCID mice under specific pathogen–free conditions [49, 50]. Cells (0.2 × 10^6^ in 100 µL) were injected intravenously (retro orbital vein). Injected mice were randomly assigned to receive intra-peritoneal MC2884 (1 mg/kg) or vehicle for two weeks (8 doses) (10 mice per group). All animal procedures were performed in accordance with protocols approved by the local Committee for Animal Experimentation and with the permission of the Italian Health Ministry (n° 626/2015). At 16 days after injection, mice were sacrificed and the femurs and spleens were collected. Bone marrow (BM) and spleen cells were analyzed by FACS. To avoid non-specific stain, cells were mixed with mouse IgG and incubated with indicated antibodies for 30’. The mixture was depleted of erythrocytes and fixed (BD Pharmingen). NB4 and U937-AML cells were examined with FITC-anti-human CD45 antibody (eBioscence) and APC-conjugated anti-mouse CD45 antibody (Miltenyi). NB4-luc xenografts were performed by using a clone of NB4 luciferase transduced and by injecting 1 × 10^6^ cells (IP). Injected mice were randomly assigned to receive intra-peritoneal MC2884 (10 mg/kg) or vehicle for 4 weeks (every second day for the first 2 weeks; every third day for the rest). Tumor growth was monitored by weekly bioluminescence imaging (BLI) acquisitions using an IVIS 3D Imaging System (Caliper Alameda, USA). To quantify bioluminescence, the integrated fluxes of photons (photons per s) within each area of interest were determined using the Living Images Software Package 3.2 (Caliper, Alameda, USA). Emission data were collected and normalized to bioluminescence of the injection day. Efficacy of drug treatment was assessed as inhibition of BLI emission comparing those MC2884 treated mice group and vehicle groups.

### Colon cancer xenografts

HCT116 cell line was grown in McCoy’s medium, supplemented with 10% inactivated FBS, 2 mM glutamine and standard concentration of antibiotics. 7- to 8-week-old male CD1 nude athymic mice were purchased from Charles River and under pathogen-free conditions in accordance with European Directives no. 2010/63 and with Italian D.L. 26/2014. Mice were injected subcutaneously into the right flank with 4 × 10^6^ of HCT116 cells; tumor volume (mm^3^) was evaluated three times a week by caliper using the formula *D*_*max*_ *x d*_*min*_*^*^*2*^*/2*, where ‘d’ and ‘D’ are the shortest and the longest diameters, respectively. For all experiments, mice were randomly divided (*n* = 7) and intra-peritoneal injected by day five every day with: vehicle sesame oil/DMSO 6%; MC2884 10 mg/Kg in sesame oil/DMSO 6%. Mice were sacrificed after 20 days of drug treatment and tumors explanted for immunohistochemical analyses. For HCT116-DKO colon cancer [51], 6 × 10^6^ cells were injected.

### Histology, Immunohistochemical analyses and TUNEL assay *in vivo* (xenograft)

The biopsy specimens were fixed in 10% buffered-formalin and paraffin-embedded. Sections of 5 μM were stained with haematoxylin-eosin, and haematoxylin-van Gieson. For immunohistochemistry, specimens were incubated in a microwave oven for 15 min in 10 mM buffered citrate pH 6.0 followed by the immunohistochemical procedure for Ki67 (Santa Cruz Biotechnology Inc., CA, USA), H3K27me3, H3K9/14ac (Diagenode). The conventional avidin-biotin complex procedure was applied according to manufacturer’s protocol (Dako Carpinteria, CA, USA) and incubated with secondary antibody. Positive staining was revealed by DAB chromogen, according to supplier’s instructions followed by counterstaining with Mayer’s hematoxylin. Slides were cover-slipped with a xylene-based mounting medium. Staining was scored as percentage of positive nuclei per high power field 10 × 40. Negative controls for each tissue section were performed omitting the primary antibody. Positive controls included in each experiment consisted of tissue previously shown to express the antigen of interest. TUNEL reaction was performed using the peroxidase-based Apoptag kit (Oncor, Gaithersburg, MD, US). TUNEL positive cells were detected with DAB and H_2_O_2_ according to the supplier’s instructions. The experiments were repeated on different sections for each specimen (two to four). For all immunohistochemical markers, 100 random fields (250×) per section were analysed (12.5 mm^2^). Mann–Whitney and Wilcoxon tests were used to assess the relationship between ordinal data. Two-tailed *P* value was considered significant when ≤ 0.05. SPSS software (version 10.00, SPSS, Chicago, IL, USA) was used for statistical analysis.

### Chromatin immunoprecipitation (ChIP)

ChIP extracts preparation and procedures have been carried out following IHEC procedures using Diagenode antibodies and as reported [48, 52, 53]. The fold enrichment of H3K27ac and H3K27me3 ChIP’ed DNA was evaluated. Primer sequences were as follows: BCL2 promoter region (at -410 and -282 from +1), forward (5′- GTG TTC CGC GTG ATT GAA GAC-3′) and reverse (5′- CAG AGA AAG AAG AGG AGT TAT AA-3′); for chr21:44496353–44496918/CBS region, forward (5′- CGC AGA ACA GTC GCC TTG-3′) and reverse (5′- GTC CAG AGC ACG ATG TTT GG-3′); for chr17:4938577–4939058/SLC52A1 region, forward (5′- CGA GTT GGA GAG GGG AGT G-3′) and reverse (5′- AAC AAA ACC CCA GCT GTG TG-3′).

### ChIP-sequencing

For ChIP-seq peak calling the BAM files were first filtered to remove the reads with mapping quality less than 15, followed by fragment size modeling (http://code.google.com/p/phantompeakqual-tools/). The peak-calling algorithm MACS2 (http://github.com/taoliu/MACS/) was used to detect the binding sites for the three studied histone marks at default *q*-value (5.00e-02). H3K27ac peaks were called using the default (narrow) setting. H3K27ac tracks in mouse APL cells and human HCT116 cells were analyzed similarly.

The intensity graphs were generated using the in house script makeColorprofiles.pl. Discriminating hyperacetylated regions were identified using DESEQ (http://bioconductor.org/packages/release/bioc/html/DESeq.html). Box plots were generated using the normalized read counts (corrected for sequencing depth and region length) and DESEQ was used to calculate for statistical significant differences. For comparisons either the Welch *T*-test or the Mann Whithey *U* test were used. Functional analysis was performed using GREAT: http://great.stanford.edu/public/html/.

### Primary samples

Blasts cells were purified by the Ficoll-Hypaque gradient separation method (GE Healthcare). RNA was obtained by using TRIzol (Life Technologies) according to the manufacturer’s recommendations. Isolation of genomic DNA has complied the FlexiGene DNA protocol (Qiagen).

ChIP extracts preparation has been carried out following IHEC procedures and as reported in [47, 48, 53, 54].

### Tet2^−/−^APL experiments

MSCV backbone was used to express human PML-RARA fusion (a kind gift from H De Thé) and used to transduce marrow progenitors from 5FU-treated 5-week-old mice wild type or inactivated for Tet2. After engraftment in irradiated recipient and malignant development, leukemic cells were grown *in vitro*, in standard M3434 methylcellulose conditions. For the experiments, cells were transferred in liquid culture and treated with MC2884. After 16 h of treatment, apoptotic cells were stained with Annexin V/APC and 7-AAD (Beckton Dickinson) and analyzed on a FACS CantoII (Beckton Dickinson). FACS data were analyzed by FlowJo Software (v8.8.7).

### Patient characteristics

**Table:** 

Number	Blueprint ID	Leukemia	karyotype
pt#1	n/a	AML	NK
pt#2	n/a	AML	NK
pt#3	n/a	AML	del(2)
pt#4	n/a	AML	+8
pt#5	n/a	AML	+8
pt#6	n/a	AML	inv3
pt#7	284	APL	t(15;17)
pt#8	289	APL	t(15;17)
pt#9	302	APL	t(15;17)
pt#10	n/a	ALL	

## SUPPLEMENTARY MATERIALS FIGURES AND TABLES




