# Supplementary material for: Combined HAT/EZH2 modulation leads to cancer-selective cell death
Source: Oncotarget. 2018 May 22;9(39):25630–46. doi: 10.18632/oncotarget.25428 (PMC5986654; doi:10.18632/oncotarget.25428)
Supplement: Supplementary file 1 [file oncotarget-09-25630-s001.pdf]

# Combined HAT/EZH2 modulation leads to cancer-selective cell death

## SUPPLEMENTARY MATERIALS

### Chemistry

The compounds MC2886, MC2911, MC2912, MC2913, MC2914, MC2908, MC2884, MC2909 and MC2910 were prepared through aldol condensation between the cyclic ketones and the properly substituted benzaldehyde in presence of barium hydroxide octahydrate and methanol at room temperature (Schemes 1, 2). By performing the condensation like in scheme 1 and 2 the 3,5-bis-(3-bromobenzylidene)piperidin-4-one scaffold was synthesized (Scheme 3), afterwards it underwent *N*-alkylation with corresponding alkyl chloride by using anhydrous potassium carbonate in acetonitrile at 60° C for providing MC3269, MC3272 and MC3207 (Scheme 3). Differently, MC3146 and MC3187 were prepared by treating 3,5-bis-(3-bromobenzylidene)piperidin-4-one with appropriate acyl chlorides in presence of triethylamine and dichloromethane at 0° C. (Scheme 3). Only for derivative MC3395 the aldol condensation was performed by using 4N hydrochloric acid in ethanol refluxing for 2 days (Scheme 4)[1–4].

### Scheme 1[a]

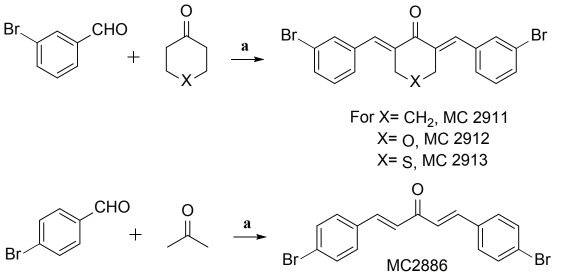


### Scheme 2[a]

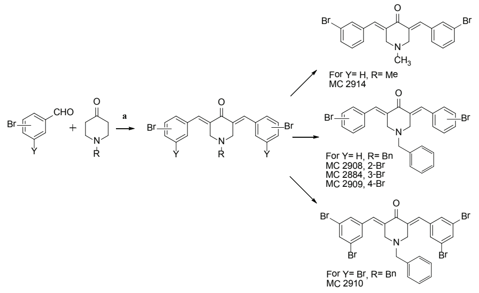


### Scheme 3[a]

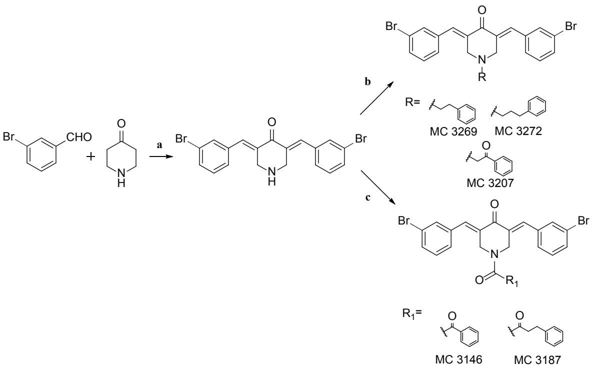


### Scheme 4[a]

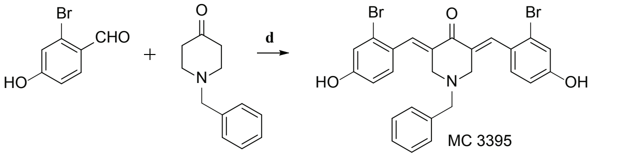


[a]*Reagents and conditions* (a) $Ba(OH)_2 \times 8\ H_2O$, $CH_3OH$, r.t., 2 h; b) alkyl bromide, $K_2CO_3$, $CH_3CN$, 60° C, 2 h; c) $Et_3N$, acyl chloride, DCM, 0° C, 1 h; d) 4N HCl, ethanol, 100° C, 2 days.

Melting points were determined on a Buchi 530 melting point apparatus and are uncorrected. $^1$H NMR and $^{13}$C NMR spectra were recorded at 400 and 100 MHz, respectively, on a Bruker AC 400 spectrometer; chemical shifts are reported in δ (ppm) units relative to the internal reference tetramethylsilane ($Me_4Si$). EIMS spectra were recorded with a Fisons Trio 1000 spectrometer; only molecular ions ($M^+$) and base peaks are given. All compounds were routinely checked by TLC and 1H NMR. TLC was performed on aluminum-backed silica gel plates (Merck DC, Alufolien Kieselgel 60 F254) with spots visualized by UV light. All solvents were reagent grade and, when necessary, were purified and dried by standard methods. Concentration of solutions after reactions and extractions involved the use of a rotary evaporator operating at reduced pressure of ca. 20 Torr. Organic

solutions were dried over anhydrous sodium sulfate. Elemental analysis has been used to determine purity of the described compounds, that is, >95%. Analytical results are within ±0.40% of the theoretical values. All chemicals were purchased from Sigma-Aldrich, Milan (Italy), or from Alfa Aesar, Karlsruhe (Germany), and were of the highest purity.

## General procedure for the synthesis of MC2908, MC2884, MC2886, MC2909, MC2910, MC 2911, MC2912, MC2013, MC2914

### Example: synthesis of 1-benzyl-3,5-bis(3-bromobenzylidene)piperidin-4-one (MC 2884)

1-benzylpiperidin-4-one (1.06 mmol, 0.2 mL) was added to a suspension of barium hydroxide octahydrate (4.24 mmol, 1.34 g) in methanol (10 mL), and the mixture was stirred for 5 min. Then a solution of 3- bromo-benzaldehyde (2.12 mmol, 0.39 g) in methanol (10 mL) was added, and the resultant mixture was stirred at room temperature. After 2 hours water was added and the resulting suspension was filtered, the precipitate was washed with water (3 × 10 mL), dried and recrystallized to afford the pure product. $^{1}$H-NMR (DMSO-$d_6$) $\delta_H$/ppm: 3.71 (s, 2H, PhC$H_2$), 3.81 (s, 4H, N(C$H_2$)$_2$, 7.19 (s, 5H, benzene protons), 7.37–7.45 (m, 4H, benzene protons), 7.60–7.65 (m, 6H, benzene protons and PhC*H* = CCO); $^{13}$C-NMR (DMSO-$d_6$) $\delta_C$/ppm: 53.4 (2C), 64.4, 123.0 (2C), 127.2, 127.5 (2C), 128.4 (2C), 128.8 (2C), 129.6 (2C), 130.8 (2C), 132.6, 132.7 (2C), 136.1 (2C), 140.6 (2C), 145.9 (2C), 186.0; MS (EI): *m/z* [M+H]$^+$: 523.46.

## (1E,4E)-1,5-bis(4-bromophenyl)penta-1,4-dien-3-one (MC 2886)

$^{1}$H-NMR (DMSO-$d_6$) $\boldsymbol{\delta}_H$/ppm: 7.36–7.40 (d, 2H, PhC*H* = CHCO), 7.67–7.80 (m, 10H, benzene protons and PhCH = C*H*CO); $^{13}$C-NMR (DMSO-$d_6$) $\boldsymbol{\delta}_C$/ppm: 122.5 (2C), 123.4 (2C), 128.7 (4C), 131.6 (4C), 134.4 (2C), 142.1 (2C), 188.8; MS (EI): *m/z* [M+H]$^+$: 389.93.

## 1-benzyl-3,5-bis(2-bromobenzylidene)piperidin-4-one (MC 2908)

$^{1}$H-NMR (DMSO-$d_6$) $\boldsymbol{\delta}_H$/ppm: 3.60 (s, 2H, PhC$H_2$), 3.71 (s, 4H, N(C$H_2$)$_2$, 7.16 (s, 5H, benzene protons), 7.30–7.42 (m, 7H, benzene protons), 7.73–7.75 (m, 4H, benzene protons and PhC*H* = CCO); $^{13}$C-NMR (DMSO-$d_6$) $\boldsymbol{\delta}_C$/ppm: 53.4 (2C), 64.4, 125.2 (2C), 127.0 (2C), 127.2, 127.5 (2C), 127.6 (2C), 128.4 (2C), 128.8 (2C), 132.6 (3C), 134.3 (2C), 140.6 (2C), 145.9 (2C), 186.0; MS (EI): *m/z* [M+H]$^+$: 522.70.

## 1-benzyl-3,5-bis(4-bromobenzylidene)piperidin-4-one (MC 2909)

$^{1}$H-NMR (DMSO-$d_6$) $\boldsymbol{\delta}_H$/ppm: 3.71 (s, 2H, PhC$H_2$), 3.80 (s, 4H, N(C$H_2$)$_2$, 7.20–7.25 (m, 5H, benzene protons), 7.39 (m, 4H, benzene protons), 7.58–7.64 (m, 6H, benzene protons and PhC*H* = CCO); $^{13}$C-NMR (DMSO-$d_6$) $\boldsymbol{\delta}_C$/ppm: 53.0 (2C), 57.8, 124.0 (2C), 125.3, 126.8 (2C), 128.3 (2C), 130.7 (4C), 133.0 (4C), 134.9, 136.3 (2C), 138.9 (2C), 141.7 (2C), 187.4; MS (EI): *m/z* [M+H]$^+$: 523.78.

## 1-benzyl-3,5-bis(3,5-dibromobenzylidene)piperidin-4-one (MC 2910)

$^{1}$H-NMR (DMSO-$d_6$) $\boldsymbol{\delta}_H$/ppm: 3.71 (s, 2H, PhC$H_2$), 3.79 (s, 4H, N(C$H_2$)$_2$, 7.22 (s, 4H, benzene protons), 7.56 (s, 2H, benzene protons), 7.87 (s, 2H, PhC*H* = CCO); $^{13}$C-NMR (DMSO-$d_6$) $\boldsymbol{\delta}_C$/ppm: 53.4 (2C), 64.4, 123.3 (4C), 127.2, 128.4 (2C), 128.8 (2C), 128.9 (4C), 132.6, 133.9 (2C), 139.6 (2C), 140.6 (2C), 145.9 (2C), 186.0; MS (EI): *m/z* [M+H]$^+$: 680.82.

## 2,6-bis((E)-3-bromobenzylidene)cyclohexan-1-one (MC 2911)

$^{1}$H-NMR (DMSO-$d_6$) $\boldsymbol{\delta}_H$/ppm: 1.71–1.74 (m, 2H, -C$H_2$-cyclohexanone), 2.87–2.90 (t, 4H, -C$H_2$-cyclohexanone), 7.40–7.44 (t, 2H, benzene protons), 7.54–7.61 (m, 6H, benzene protons and PhC*H*-), 7.73 (s, 2H, benzene protons); $^{13}$C-NMR (DMSO-$d_6$) $\boldsymbol{\delta}_C$/ppm: 25.1, 26.3 (2C), 123.1 (2C), 127.6 (2C), 129.5 (2C), 130.9 (2C), 132.4 (2C), 132.7 (2C), 136.3 (2C), 137.2 (2C), 190.6; MS (EI): *m/z* [M+H]$^+$: 429.96.

## 3,5-bis(3-bromobenzylidene)dihydro-2H-pyran-4(3H)-one (MC 2912)

$^{1}$H-NMR (DMSO-$d_6$) $\boldsymbol{\delta}_H$/ppm: 4.91 (s, 4H, O(C$H_2$)$_2$, 7.43–7.45 (m, 4H, benzene protons), 7.65–7.69 (m, 6H, benzene protons and PhC*H* = CCO); $^{13}$C-NMR (DMSO-$d_6$) $\boldsymbol{\delta}_C$/ppm: 67.3 (2C), 123.0 (2C), 127.5 (2C), 129.6 (2C), 130.8 (2C), 132.7 (2C), 136.1 (2C), 143.8 (2C), 146.1 (2C), 186.0; MS (EI): *m/z* [M+H]$^+$: 433.93.

## 3,5-bis(3-bromobenzylidene)dihydro-2H-thiopyran-4(3H)-one (MC 2913)

$^{1}$H-NMR (DMSO-$d_6$) $\boldsymbol{\delta}_H$/ppm: 3.97 (s, 4H, O(C$H_2$)$_2$, 7.41–7.45 (t, 2H, benzene protons), 7.53–7.57 (m, 4H, benzene protons), 7.61–7.63 (d, 2H, benzene protons), 7.73 (s, 2H, PhC*H* = CCO); $^{13}$C-NMR (DMSO-$d_6$) $\boldsymbol{\delta}_C$/ppm: 30.2 (2C), 123.0 (2C), 127.5 (2C), 129.6 (2C), 130.8 (2C), 132.7 (2C), 136.1 (2C), 139.0 (2C), 146.9 (2C), 186.0; MS (EI): *m/z* [M+H]$^+$: 449.91.

### 3,5-bis(3-bromobenzylidene)-1-methylpiperidin-4-one (MC2914)

$^1$H-NMR (DMSO-$d_6$) $\delta_H$/ppm: 2.39 (s, 3H, NC*H*$_3$), 3.73 (s, 4H, (C*H*$_2$)$_2$NCH$_3$), 7.42–7.46 (m, 2H, benzene protons), 7.50–7.52 (d, 2H, benzene protons), 7.57 (s, 2H, PhC*H* = CCO), 7.62–7.64 (d, 2H, benzene protons), 7.71 (s, 2H, benzene protons); $^{13}$C-NMR (DMSO-$d_6$) $\delta_C$/ppm: 45.0, 56.1 (2C), 123.0 (2C), 127.6 (2C), 129.8 (2C), 130.8 (2C), 132.6 (2C), 136.0 (2C), 140.8 (2C), 145.9 (2C), 186.1; MS (EI): *m/z* [M+H]$^+$: 445.97.

### General procedure for the synthesis of MC3269, MC3272, MC3207

### Example: synthesis of 3,5-bis(3-bromobenzyliden)-1-(3-phenylpropyl) piperidin-4-one (MC 3272)

To a suspension of anhydrous $K_2CO_3$ (0.69 mmol, 0.954 g) in acetonitrile (10 mL), the 3,5-bis(3-bromobenzyliden)piperidin-4-one (0.46 mmol, 0.2 g) and the 3-bromo-1-phenylpropane (1.38 mmol, 0.21 mL) were added and the resulting suspension was stirred at 60° C. After 2 hours the solvent was evaporated, water (50 mL) was added and the aqueous solution was extracted with dichloromethane (3 × 30 mL). The collected organic phases were washed with a saturated solution of NaCl (30 mL) and then dried with anhydrous $Na_2SO_4$, filtered and evaporated under vacuum to afford a crude that was purified on silica gel (AcOEt/*n*-hexane 1:3) to obtain the desired compound. $^1$H-NMR (DMSO-$d_6$) $\delta_H$/ppm: 1.65–1.71 (m, 2H, -NCH$_2$C*H*$_2$CH$_2$Ph), 2.47–2.51 (t, 2H, -NCH$_2$CH$_2$C*H*$_2$Ph), 2.54–2.58 (t, 2H, -NC*H*$_2$CH$_2$CH$_2$Ph), 3.68 (s, 4H, piperidonic protons), 7.05–7.38 (m, 11H, benzene protons), 7.67–7.69 (d, 2H, benzene protons), 7.96 (s, 2H, PhC*H* = CCO); $^{13}$C-NMR (DMSO-$d_6$) $\delta_C$/ppm:27.5, 31.0, 53.8 (2C), 57.0, 125.2 (2C), 126.0, 127.0 (2C), 127.5 (2C), 127.6 (2C), 128.1 (2C), 128.8 (2C), 132.6 (2C), 134.3 (2C), 140.6 (2C), 142.0, 145.9 (2C), 186.0; MS (EI): *m/z* [M+H]$^+$: 551.41.

### 3,5-bis(3-bromobenzylidene)-1-phenethylpiperidin-4-one (MC3269)

$^1$H-NMR (DMSO-$d_6$) $\delta_H$/ppm: 2.73–2.77 (t, 2H, NCH$_2$C*H*$_2$Ph), 2.83–2.86 (t, 2H, NC*H*$_2$CH$_2$Ph), 3.87 (s, 4H, (C*H*$_2$)$_2$NCH$_2$CH$_2$Ph), 7.13–7.32 (m, 9H, benzene protons), 7.52–7.53 (m, 4H, benzene protons), 7.75 (s, 2H, PhC*H* = CCO); $^{13}$C-NMR (DMSO-$d_6$) $\delta_C$/ppm: 33.6, 53.9 (2C), 58.9, 123.1 (2C), 125.8, 127.5 (2C), 127.7 (2C), 128.8 (2C), 129.6 (2C), 130.7 (2C), 132.8 (2C), 136.3 (2C), 139.5, 140.6 (2C), 145.8 (2C), 186.2; MS (EI): *m/z* [M+H]$^+$: 536.01.

### 3,5-bis(3-bromobenzylidene)-1-(2-oxo-2-phenylethyl)piperidin-4-one (MC3207)

$^1$H-NMR (DMSO-$d_6$) $\delta_H$/ppm: 4.09 (s, 4H, (C*H*$_2$)$_2$NCH$_2$COPh), 4.11 (s, 2H, NC*H*$_2$COPh), 7.31–7.56 (m, 11H, benzene protons), 7.77 (s, 2H, PhC*H* = CCO), 7.92–7.94 (d, 2H, benzene protons); $^{13}$C-NMR (DMSO-$d_6$) $\delta_C$/ppm: 53.2 (2C), 71.0, 123.2 (2C), 127.5 (2C), 128.7 (2C), 128.8 (2C), 129.8 (2C), 131.0 (2C), 132.7 (2C), 133.2, 135.3, 136.2 (2C), 140.7 (2C), 146.2 (2C), 186.1, 195.5; MS (EI): *m/z* [M+H]$^+$: 549.99.

### General procedure for the synthesis of MC3146, MC3187

### Example: synthesis of 3,5-bis(3-bromobenzylidene)-1-(3-phenylpropanoyl) piperidin-4-one (MC3187)

To a stirring solution of 3,5-bis(3-bromobenzylidene) piperidin-4-one (0.45 mmol, 195 mg) and $Et_3N$ (0.76 mmol, 0.11 mL) in dry dichloromethane (5 mL), hydrocinnamoyl chloride (0.67 mmol, 0.1 mL) was slowly added at 0° C. The resulting mixture is then allowed to stir at room temperature. After 1 hour the reaction was quenched with water (50 mL) and extracted with dichloromethane (3 × 30 mL). The collected organic layers were washed with HCl 2N (3 × 30 mL) and then with a saturated solution of NaCl (30 mL). The organic phase was dried with anhydrous $Na_2SO_4$, filtered and evaporated under vacuum to afford a crude residue that was then purified on silica gel (AcOEt/*n*-hexane 1:3) to afford the desired product. $^1$H-NMR (DMSO-$d_6$) $\delta_H$/ppm: 2.45–2.49 (t, 2H, -COC*H*$_2$), 2.83–2.87 (t, 2H, PhC*H*$_2$), 4.57 (s, 2H, -NC*H*$_2$), 4.90 (s, 2H, -NC*H*$_2$), 6.98–7.00 (d, 2H, benzene protons), 7.18–7.63 (m, 9H, benzene protons), 7.77 (s, 2H, benzene protons); $^{13}$C-NMR (DMSO-$d_6$) $\delta_C$/ppm: 31.4, 33.6, 47.4 (2C), 123.0 (2C), 125.9, 127.5 (2C), 127.7 (2C), 128.6 (2C), 129.6 (2C), 130.8 (2C), 132.7 (2C), 136.1 (2C), 140.6 (2C), 141.3, 145.9 (2C), 172.3, 186.0; MS (EI): *m/z* [M+H]$^+$: 565.24.

#### 1-benzoyl-3,5-bis(3-bromobenzylidene)piperidin-4-one (MC3146)

$^1$H-NMR (DMSO-$d_6$) $\delta_H$/ppm: 4.68 (s, 2H, -NC*H*$_2$), 4.95 (s, 2H, -NC*H*$_2$), 7.15–7.36 (m, 9H, benzene protons), 7.50–7.86 (m, 6H, benzene protons); $^{13}$C-NMR (DMSO-$d_6$) $\delta_C$/ppm: 47.9 (2C), 123.1 (2C), 127.3 (2C), 127.7 (2C), 128.6 (2C), 129.7 (2C), 129.9, 130.9 (2C), 132.7 (2C), 135.4, 136.3 (2C), 140.7 (2C), 145.8 (2C), 169.6, 186.2; MS (EI): *m/z* [M+H]$^+$: 535.98.

#### Synthesis of 1-benzyl-3,5-bis((E)-2-bromo-4-hydroxybenzylidene)piperidin-4-one (MC3395)

A solution of 2-bromo-4-hydroxy-benzaldehyde (0.5 g, 2.49 mmol) and 1-benzyl-4-piperidone (1.24 mmol,

0.235 g) in a mixture of 4N hydrochloric acid (10 mL) and ethanol (10 mL) was refluxed at 100° C for 2 days. After this time the reaction was concentrated under vacuum, water (20 mL) was added to the residue and the formed solid was filtered and recrystallized by acetonitrile/ethanol to afford the pure MC3395 as a yellow solid. $^{1}$H-NMR (DMSO-$d_6$) $\delta_H$/ppm:

3.62 (s, 2H, PhC$H_2$-), 3.70 (s, 4H, -C$H_2$-piperidone), 6.78–6.80 (d, 2H, benzene protons), 7.11–7.73 (m, 9H, benzene protons and PhC*H*-), 7.73 (s, 2H, benzene protons); $^{13}$C-NMR (DMSO-$d_6$) $\delta_C$/ppm: 53.4 (2C), 64.4, 114.9 (2C), 119.1 (2C), 120.4 (2C), 126.9 (2C), 127.2, 128.4 (2C), 128.6 (2C), 130.2 (2C), 132.7, 140.7 (2C), 145.9 (2C), 155.7 (2C), 186.2; MS (EI): *m/z* [M+H]$^+$: 552.99.

## Surface plasmon resonance

SPR analyses were performed on a Biacore 3000 optical biosensor equipped with research-grade CM5 sensor chips (Biacore AB). Recombinant p300/KAT3B (Enzo Life Sciences, cat. # BML-SE451; GenBank accession no. NM_001429) HAT domain was immobilized (30 µg/mL in 10 mM sodium acetate, pH 4.5) were immobilized at a flow rate of 10 µL/min by using standard amine-coupling protocols to obtain densities of 15 kRU. Myoglobin was used as negative control and one flow cell was left empty for background subtractions. All compounds, dissolved in DMSO (100%), were diluted in HBS (10 mM HEPES pH 7.4, 0.15 M NaCl) always maintaining a final 0.1% DMSO concentration. Binding experiments were performed at 25° C, by using a flow rate of 30 µL/min, with 120 s monitoring of association and 300 s monitoring of dissociation. Regeneration of the surfaces was performed, when necessary, by a 10 s injection of 1 mM NaOH.

The simple 1:1 Langmuir binding fit model of the BIA evaluation software was used for determining

**Chemical and physical data of compounds MC2884, MC2908, MC2909, MC2886, MC2910, MC2914, MC3269, MC3272, MC3146, MC3207, MC3187, MC2911, MC2912, MC2913, MC3395**

| Compd | Structure | Melting point (°C) | Recrystallization solvent | Yields (%) |
|---|---|---|---|---|
| MC2884 | 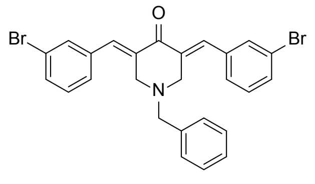 | 238–240 | Acetonitrile/ethanol | 78 |
| MC2908(1) | 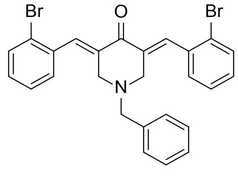 | 144–146 | benzene | 82 |
| MC2909(2) | 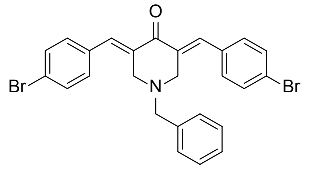 | 163–165 | Benzene/acetonitrile | 84 |
| MC2886(3) | 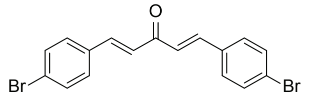 | 198–200 | Acetonitrile/ethanol | 86 |
| MC2910 | 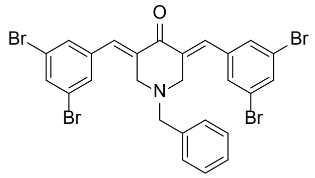 | 178–180 | Benzene/acetonitrile | 73 |
| MC2914(1) | 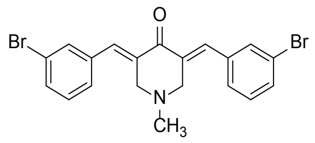 | 130–132 | Cyclohexane/benzene | 74 |

| | | | | |
|---|---|---|---|---|
| MC3269 | 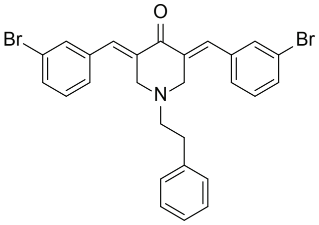 | 120–123 | Cyclohexane/Benzene | 63 |
| MC3272 | 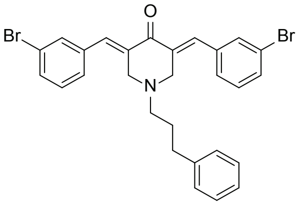 | 121–123 | Cyclohexane/Benzene | 75 |
| MC3146 | 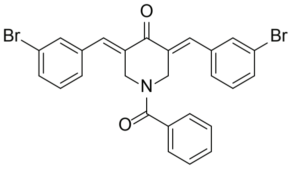 | 135–137 | Cyclohexane/Benzene | 78 |
| MC3207 | 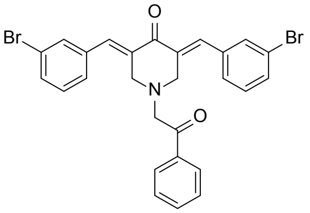 | 121–123 | Cyclohexane/Benzene | 75 |
| MC3187 | 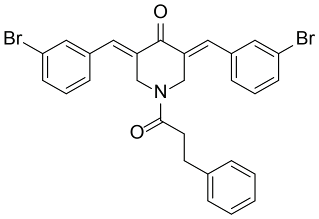 | 113–115 | Cyclohexane | 67 |
| MC2911[4] | 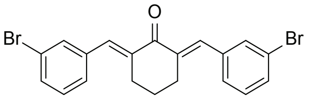 | 175–177 | Cyclohexane/Benzene | 78 |
| MC2912 | 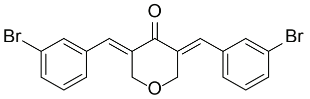 | 170–172 | Benzene/acetonitrile | 83 |
| MC2913 | 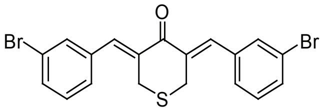 | 148–150 | Benzene | 80 |
| MC3395 | 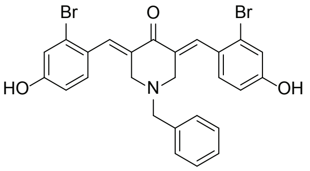 | 205–207 | Acetonitrile/ethanol | 69 |

equilibrium dissociation constants (KD) and kinetic dissociation (kd) and association (ka) constants by using equations 1 and 2:

$$dR/dt = k_a \times C \times (R_{max}-R)-k_d \times R \quad \text{Equation 1}$$

where R represents the response unit, C is the concentration of the analyte, and

$$K_D = k_d/k_a \quad \text{Equation 2}$$

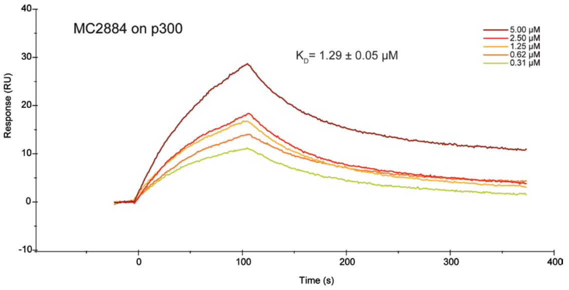


## REFERENCES


1. Rajesh SM, Bala BD, Perumal S. Multi-component, 1,3-dipolar cycloaddition reactions for the chemo-, regio- and stereoselective synthesis of novel hybrid spiroheterocycles in ionic liquid. Tetrahedron Letters. 2012; 53:5367–5371.
2. Han Z. TS, Jiang B, Yan S, Zhang X, Wu S, Hao W, Cao X, Shi F, Zhang G. An Efficient and Chemoselective Synthesis of 1,6-Naphthyridines and Pyrano[3,2-c]pyridines under Microwave Irradiation. Synthesis. 2009:8.
3. Wei X, Du ZY, Zheng X, Cui XX, Conney AH, Zhang K. Synthesis and evaluation of curcumin-related compounds for anticancer activity. Eur J Med Chem. 2012; 53:235–245.
4. Wan YW, Chen X, Pang L, Ma R, Yue C, Yuan R, et al. Synthesis and Fluorescence Properties of α,α′-Bis(substituted-benzylidene)cycloalkanones Catalyzed by 1-Methyl-3(2-(sulfooxy)ethyl)-1H-imidazol-3-ium Chloride. Synthetic Communications. 2010; 40:9.


## SAR STUDIES

When tested in leukemia NB4 cells at 3 µM for 30 h, MC2884 induced 52.1% cell death. Some MC2884 analogues have been tested to obtain SAR data (Supplementary Table 1). In particular, the shift of bromine atoms from *meta* to *ortho* (MC2908) and mainly to *para* (MC2909) position at the benzene rings reduced cell death to 30.3% and to 8.8%, respectively. Also the introduction of further bromine atoms at the *meta* positions of the two phenyl rings (MC2910) abated cell death induction (4.2%) of the derivative. At the N1 position, replacement of the benzyl with a methyl group (MC2914) led to a decrease of cell death induction (36%), whereas introduction of the longer phenylethyl moiety (MC3269) gave an increase of the effect (62.5% cell death). Further stretching of the N1 substituent to the phenylpropyl one (MC3272) lowered cell death induction (29.2%). Changing the N1-benzyl group of MC2884 with the N1-benzoyl group (MC3146) produced a severe loss of activity (13.2% cell death), but it was partially restored by introducing at N1 a 1-phenyl-2-ethan-1-one (MC3207, 38% cell death) or 3-phenyl-1-propan-1-one (MC3187, 28.9% cell death) unit, thus giving more flexibility to the N1 substituent. Replacement of the N1-benzyl-4-piperidone moiety with the tetrahydro-4*H*-pyran-4-one (MC2912) totally abated the cellular effect of the derivative (4.1% cell death), whereas the use of the isosteric tetrahydro-4H-thiopyran-4-one (MC2913) strongly restored the cell death, MC2913 showing the same potency of MC2884 (52.7% cell death).

Western blot analyses (and their quantification by ImageJ) performed on NB4 cells to detect the levels of acetyl-H3K9-14 (as a marker of HAT/anti-HAT activity) and H3K27me3/2 (as marker of PRC2/EZH2 activity) showed a decrease of histone acetylation and methylation after treatment with 3 µM compounds for 48 h, thus confirming the role of dual HAT/EZH2 inhibitors for these derivatives. In addition, the tested compounds also lowered the level of the EZH2 protein (SAR Figure 1).

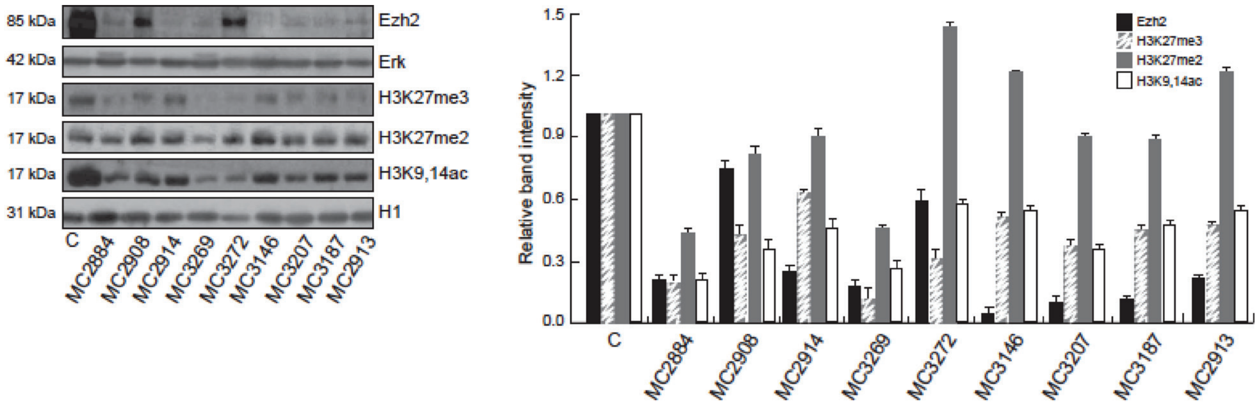


**SAR-Figure 1: Analysis of histone & non-histone target modulation by MC2884 and analogues in NB4 cells.**

**SAR-Table 1: Percentage of cell death induction by MC2884 and its analogues in NB4 and SHSY5Y cells**

| Cmpd | Structure | % Cell death | |
|---|---|---|---|
| | | NB4 cells, 3 µM, 30 h | SHSY5Y cells, 3 µM, 48 h |
| Vehicle | | 2.5 | 1.3 |
| MC2884 | 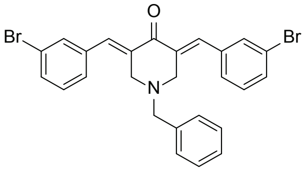 | 52.1 | 19.1 |
| MC2908 | 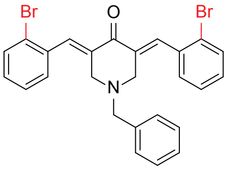 | 30.3 | 9.2 |
| MC2909 | 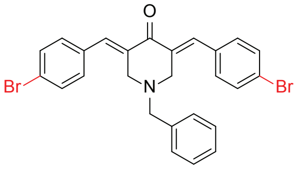 | 8.8 | 4.7 |
| MC2886 | 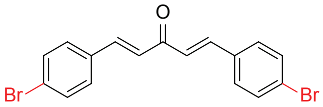 | 0,56 | 1,2 |
| MC2910 | 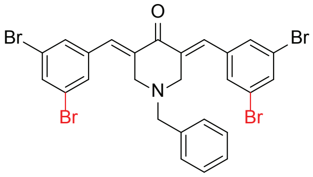 | 4.2 | 2.4 |
| MC2914 | 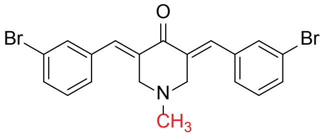 | 36.0 | 9.0 |
| MC3269 | 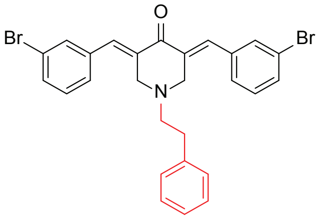 | 62.5 | 19.2 |
| MC3272 | 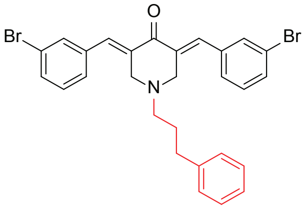 | 29.1 | 7.0 |

| | | | |
|---|---|---|---|
| MC3146 | 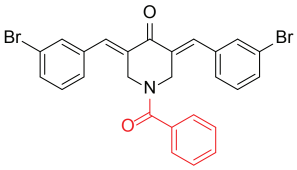 | 13.2 | 5.6 |
| MC3207 | 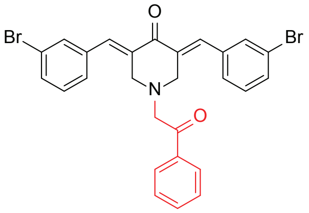 | 38.0 | 7.0 |
| MC3187 | 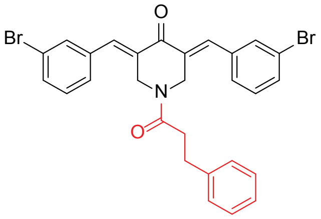 | 28.9 | 19.9 |
| MC2911 | 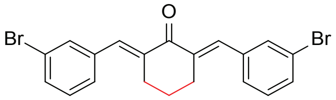 | 0,76 | 0,8 |
| MC2912 | 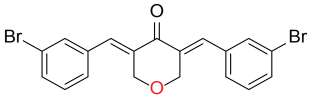 | 4.1 | 1.0 |
| MC2913 | 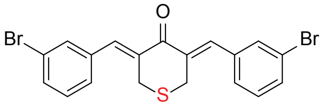 | 52.7 | 23.9 |
| MC3395 | 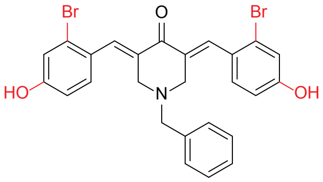 | 0,53 | 1,0 |

When tested in neuroblastoma SHSY5Y cells at 3 µM for 48 h, MC2884 and its analogues furnished similar trend of death induction as in NB4 cells (Supplementary SAR Table 1). In general, the SHSY5Y cell line is less sensitive to the tested molecules than NB4 cells. Anyway, also in this cell line the bis 2-bromophenyl (MC2908), bis 4-bromophenyl (MC2909), and bis 3,5-dibromophenyl (MC2910) were less potent or not active as cell death inducers. As in NB4 cells, substitution at N1 with methyl (MC2914) or 3-phenylpropyl (MC3272) groups reduced the potency of the corresponding derivatives whereas introduction of a phenethyl group (MC3269) gave a compound with the same potency as MC2884. The presence of a carbonyl function within the N1 substituent abated the activity of the compounds, with the exception of that showing a 3-phenyl-1-propan-1-one chain at N1 (MC3187), which in this case was slightly more potent than MC2884. The tetrahydro-4*H*-pyran-4-one MC2912 failed in inducing cell death in SHSY5Y cells, whereas its thio-analogue MC2913 displayed the best death induction activity, similarly to what observed in NB4 cells.

Interestingly, compounds inactive in inducing cell death in both cell lines such as MC2910, 2912, 2909, 2886, 2911, 3395 where also unable to modulate histone acetylation and EZH2 deregulation (SAR Figure 2). In full agreement, only MC2884 was able to down-regulate BCL2 expression levels at both RNA (histogram bars) and protein levels (western blot) suggesting that this action is crucial for the induction of apoptosis.

Finally, to causally connect the anticancer action with the dual p300 and EZH2 targeting of the drug, we evaluated MCF7 cells viability after overexpression of WT or catalytic mutants of p300 or EZH2, after treatment with MC2884 (SAR Figure 3). Interestingly, expression of p300 or EZH2 to higher levels leaded to increased cell death after MC2884, suggesting that

both targets are important for the anticancer action of the drug in these settings. When catalytic mutants where used, only the mutant p300 expression fully reversed the MC2884 induction of cell death, whereas the mutant EZH2 gave rise to a partial reversion, potentially suggesting that both activities are needed but p300 inhibitory enzymatic action might be prevalent. Note that MC2884 action on EZH2 expression levels (and thus action that might be additional to the enzymatic regulation) is also potentially playing a role.

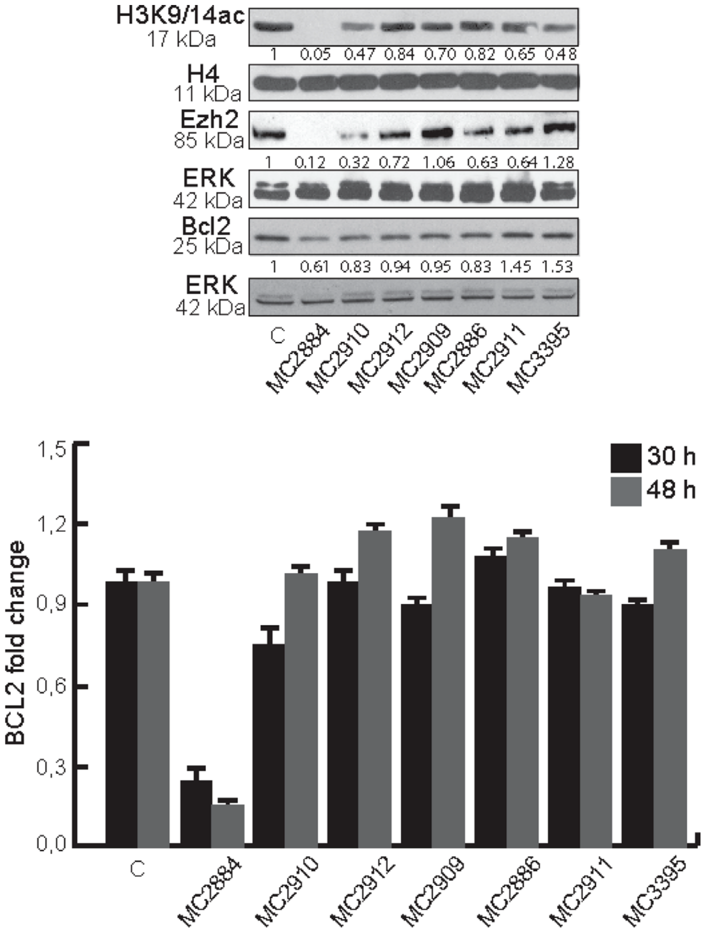


**SAR-Figure 2: Up. Evaluation of modulation of histone and non-histone targets of MC2884 and its anticancer inactive analogues in NB4 cells. Bottom. qPCR for BCL2 expression at the indicated time points.**

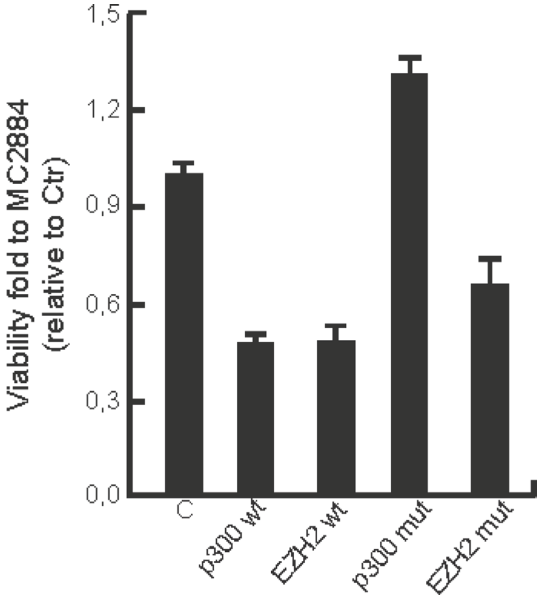


**SAR-Figure 3: Both overexpression of p300 and EZH2 improves MC2884 anticancer action; the expression of their catalytic mutants instead, reduces MC2884 induction of cell death.**

# PHARMACOLOGY

## Pharmacokinetic analysis (Mice–IV, IP PK study)

Compound was administered both intravenously and intraperitoneally to mice. Blood samples were collected at 8 time points over 24 hr and plasma analysed by LC-MS/MS to determine the concentration of compound.

The plasma time concentration profile was calculated with the main PK parameters (Co, AUClast, t.,VD, and CL).

## Formulation

For ip dosing. After formulation assessment, MC2884 was dissolved at 0.2 mg/mL. This provided a dose of 2 mg free base material/kg when administered ip in a 10 mL/kg dosing volume.

For iv dosing. Compound was dissolved at 0.4 mg/mL. This provided a dose of 2 mg free base material/kg when administered iv in a 5 mL/kg dosing volume.

## Mouse terminal IP PK study

Compound MC2884 was given ip at a dose of 2 mg/kg to a group of 24 normally fed male CD1 mice in a 10 mL/kg injection volume. Terminal blood samples (> 230 µL) were taken under CO2 from groups of 3 mice at each of 8 time-points post dose (0.083, 0.25, 0.5, 1, 2, 4, 8 and 24 h) and placed into heparinized tubes. Samples were placed on ice for no longer than 30 min before centrifugation (10,000 rpm × 3 min). Plasma samples (>100 µL) were collected into fresh tubes and frozen on dry ice. All samples were stored at −20° C

Number of plasma samples = 24.

| Average | animal(s) ,1,2,3 | | | | | | | | |
|---|---|---|---|---|---|---|---|---|---|
| Time (hr) | Pre-Dose | 0,08 | 0,25 | 0,50 | 1,00 | 2,00 | 4,00 | 8,00 | 24,00 |
| Conc (µg/mL) | | 0,401 | 0,639 | 4,589 | 7,796 | 5,146 | 0,453 | 0,109 | 0,030 |
| Std Dev | | 0,0443 | 0,4703 | 4,2551 | 1,4517 | 4,4802 | 0,4222 | 0,1082 | 0,0004 |
| %CV | | 11% | 74% | 93% | 19% | 87% | 93% | 99% | 1% |
| # Excluded Points | | | | | | | | | |
| # Tailpoints | 3 | | | | | | | | |

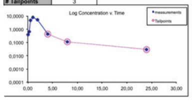


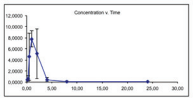


| | |
|---|---|
| Av. Weight (g) | 25 |
| Dose (mg) | 0,05 |
| Cmax (µg/mL) | 7,796 |
| C0 (µg/mL) | |
| Clast (µg/mL) | 0,030 |
| tmax (hr) | 1,00 |
| tlast (hr) | 24,00 |
| AUClast (µg-hr/mL) | 18,162 |
| AUCinf (µg-hr/mL) | 18,413 |
| t1/2 (hr) | NA |
| λz (1/hr) | 0,12 |
| MRTinf (hr) | 2,73 |
| Total CL (mL/hr) | NA |
| Total CL (mL/min/kg) | NA |
| VSS (mL) | NA |
| VZ (mL) | NA |

## Bioavailability

**AUC (IV)**

| Compound | Average (µg-hr/mL) |
|---|---|
| MC2884 | 13,322 |

**Dose (IV)**

| Compound | Average (mg) |
|---|---|
| MC2884 | 0,051 |

**AUC (IP)**

| Compound | Average (µg-hr/mL) |
|---|---|
| MC2884 | 18,413 |

**Dose (IP)**

| Compound | Average (mg) |
|---|---|
| MC2884 | 0,050 |

**% Bioavailability**

| Compound | Dose Route | Time frame (hr) | % Bioavailability |
|---|---|---|---|
| MC2884 | IP | 0-inf | 140,1% |

## Mouse terminal IV PK study

Compound MC2884 was given iv via the lateral tail vein at a dose of 2 mg/kg to a group of 24 normally fed male CD1 mice in a 5 mL/kg injection volume. Terminal blood samples (> 230 µL) were taken under CO2 from groups of 3 mice at each of 8 time-points post dose (0.083, 0.25, 0.5, 1, 2, 4, 8 and 24 h) and placed into heparinized tubes. Samples were placed on ice for no longer than 30 min before centrifugation (10,000 rpm × 3 min). Plasma samples (>100 µL) were collected into fresh tubes and frozen on dry ice. Number of plasma samples = 24.

Consistent IV profiles were obtained, with little variability across the time-points (the average profile is shown in the second spreadsheet). An average half-life of 4.58 h was calculated.

| Average | animal(s) ,1,2,3 | | | | | | | | |
|---|---|---|---|---|---|---|---|---|---|
| Time (hr) | Pre-Dose | 0,10 | 0,25 | 0,52 | 1,00 | 2,03 | 4,02 | 8,03 | 24,02 |
| Conc (µg/mL) | | 29,456 | 4,389 | 1,787 | 0,766 | 0,322 | 0,225 | 0,093 | 0,010 |
| Std Dev | | 18,9115 | 0,9747 | 0,7734 | 0,0594 | 0,0172 | 0,0332 | 0,0116 | 0,0041 |
| %CV | | 64% | 22% | 43% | 8% | 5% | 15% | 12% | 42% |
| # Excluded Points | | | | | | | | | |
| # Tailpoints | 3 | | | | | | | | |

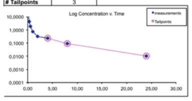


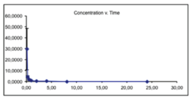


| | |
|---|---|
| Av. Weight (g) | 25 |
| Dose (mg) | 0,05 |
| Cmax (µg/mL) | |
| C0 (µg/mL) | 104,802 |
| Clast (µg/mL) | 0,010 |
| tmax (hr) | NA |
| tlast (hr) | 24,02 |
| AUClast (µg-hr/mL) | 13,256 |
| AUCinf (µg-hr/mL) | 13,322 |
| t1/2 (hr) | 4,58 |
| λz (1/hr) | 0,15 |
| MRTinf (hr) | 1,25 |
| Total CL (mL/hr) | 3,80 |
| Total CL (mL/min/kg) | 2,54 |
| VSS (mL) | 4,74 |
| VZ (mL) | 25,12 |

## Cytochrome P450 inhibition (5 Isoform IC50 determination)

ADME-Tox Cytochrome P450 inhibition of MC2884 using human liver microsomes as an estimation of *in vitro* metabolism.

MC2884 (0.1 µM–25 µM) was incubated with human liver microsomes and NADPH in the presence of a cytochrome P450 isoform-specific probe substrate. For the CYP2C9, CYP2C19, CYP2D6 and CYP3A4 specific reactions, the metabolites were monitored by mass spectrometry. CYP1A activity was monitored by measuring the formation of a fluorescent metabolite. A decrease in the formation of the metabolite compared to the vehicle control was used to calculate an IC50 value (test compound concentration which produces 50% inhibition).

## CYP1A inhibition

Six test compound concentrations (0.1, 0.25, 1, 2.5, 10, 25 µM in DMSO; final DMSO concentration = 0.3%) were incubated with human liver microsomes (0.25 mg/mL) and NADPH (1 mM) in the presence of the probe substrate ethoxyresorufin (0.5 µM) for 5 min at 37° C. The selective CYP1A inhibitor, alpha-naphthoflavone, was screened alongside the test compounds as a positive control.

## CYP2C9 inhibition

Six test compound concentrations (0.1, 0.25, 1, 2.5, 10, 25 µM in DMSO; final DMSO concentration = 0.25%) were incubated with human liver microsomes (1 mg/mL) and NADPH (1 mM) in the presence of the probe substrate tolbutamide (120 µM) for 60 min at 37° C. The selective CYP2C9 inhibitor, sulphaphenazole, was screened alongside the test compounds as a positive control.

## CYP2C19 inhibition

Six test compound samples at concentrations of 0.1, 0.25, 1, 2.5, 10, 25 μM in DMSO (final DMSO concentration = 0.25%) were incubated with human liver microsomes (0.5 mg/mL) and NADPH (1 mM) in the presence of the probe substrate mephenytoin (25 μM) for 60 min at 37° C. The selective CYP2C19 inhibitor, tranylcypromine, was screened alongside the test compounds as a positive control.

## CYP2D6 inhibition

Six test compound samples at concentrations of 0.1, 0.25, 1, 2.5, 10, 25 μM in DMSO (final DMSO concentration = 0.25%) were incubated with human liver microsomes (0.5 mg/mL) and NADPH (1 mM) in the presence of the probe substrate dextromethorphan (5 μM) for 5 min at 37° C. The selective CYP2D6 inhibitor, quinidine, was screened alongside the test compounds as a positive control.

## CYP3A4 inhibition

Six test compound concentrations (0.1, 0.25, 1, 2.5, 10, 25 μM in DMSO; final DMSO concentration 0.26%) were incubated with human liver microsomes (0.1 mg/mL) and NADPH (1 mM) in the presence of the probe substrate midazolam (2.5 μM) for 5 min at 37° C. The selective CYP3A4 inhibitor, ketoconazole, was screened alongside the test compounds as a positive control. For the CYP1A incubations, the reactions were terminated by methanol, and the formation of the metabolite, resorufin, was monitored by fluorescence (excitation wavelength = 535 nm, emission 5 wavelength = 595 nm). For the CYP2C9, CYP2C19, CYP2D6 and CYP3A4 incubations, the reactions were terminated by methanol. The samples were then centrifuged, and the supernatants were combined, for the simultaneous analysis of 4-hydroxytolbutamide, 4-hydroxymephenytoin, dextrorphan, and 1-hydroxymidazolam plus internal standard by LC-MS/MS. Formic acid in deionized water containing internal standard (final formic acid concentration = 0.1%) was added to the final sample prior to analysis. A decrease in the formation of the metabolites compared to vehicle control was used to calculate an IC50 value (test compound concentration which produces 50% inhibition).

a) CYP1A no inhibition, IC50 (μM) >25
b) CYP2C9 no inhibition, IC50 (μM) >25
c) CYP2C19 no inhibition, IC50 (μM) >25
d) CYP2D6 no inhibition, IC50 (μM) >25
d) CYP3A4 45% inhibition, observed only at 25 μM

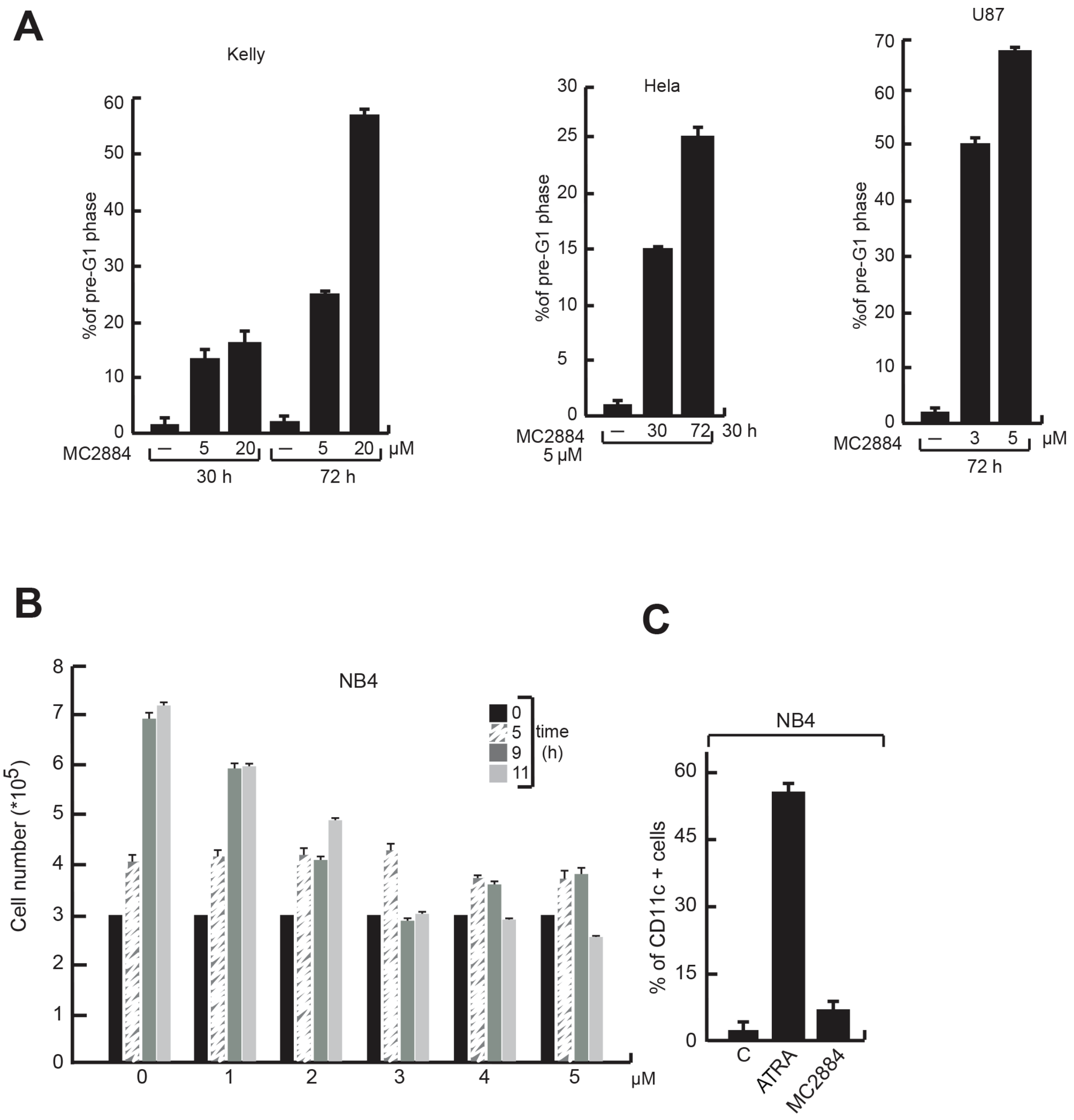


**Supplementary Figure 1: MC2884 anti-cancer effects.** (**A**) Cell death induced by MC2884 in Kelly, Hela and U87 cancer cells. (**B**) Anti-proliferative action of MC2884 in NB4 APL leukemia cells measured in real time. (**C**) Differentiation potential of MC2884 in NB4 cells at 30 hrs of treatment. ATRA was used as positive control.

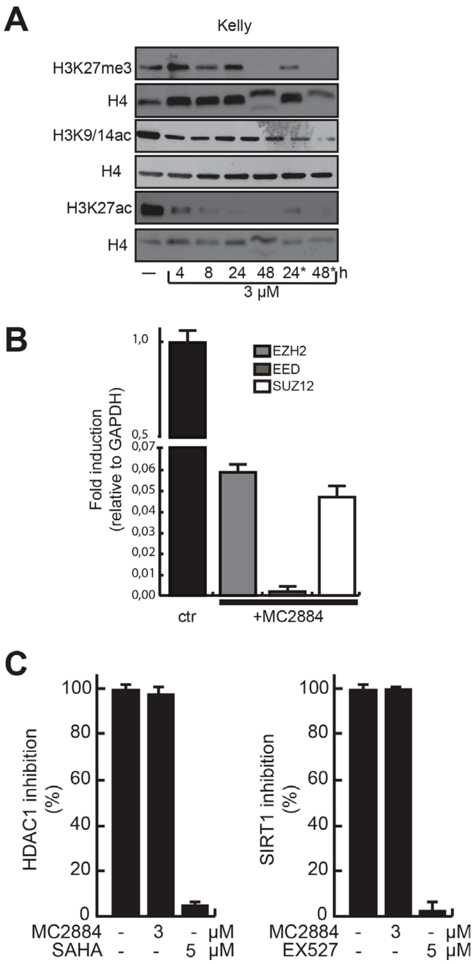


**Supplementary Figure 2: Epigenetic action of MC2884.** (**A**) H3K27me3, H3K9/14ac and H3K27ac upon MC2884 in Kelly cells. (**B**) EZH2, EED and SUZ12 RNA expression upon MC2884 induction in NB4 cells. (**C**) HDAC1 and SIRT1 *in vitro* assays upon MC2884 induction. SAHA and EX527 have been used as positive controls.

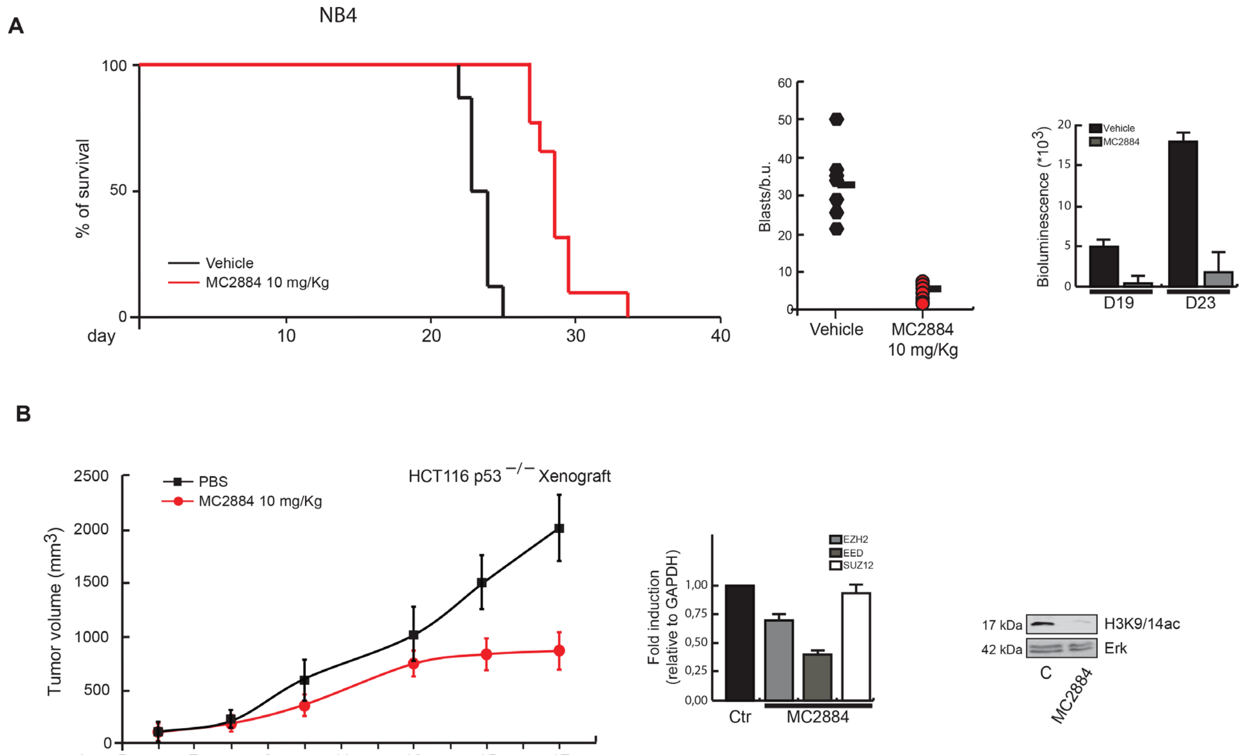


**Supplementary Figure 3: *In vivo* quantification of the anticancer action of MC2884 in xenograft models.** (**A**) NB4 luciferase expressing cells. (**B**) HCT116p53−/− colon cancer.

**Supplementary Items 1: GO analysis.** See Supplementary GO Items_1
